# Weather-dependent effects of Alternate Wetting and Drying (AWD) irrigation on ratoon rice: a 3-year field study

**DOI:** 10.1186/s12870-026-09118-0

**Published:** 2026-06-02

**Authors:** Haiwei Zhang, Youqin Xiao, Zhanzhi Gong, Nan Xu, Ruoting Mei, Xinyi Gu, Juan Li, Jiancheng Wen, Shuochen Jiang

**Affiliations:** 1 Rice Research Institute, Yunnan Agricultural University, Kunming, 650500 China; 2 College of Agriculture, Yangtze University, Jingzhou, 434025 China; 3 Key Laboratory of Crop Physiology and Ecology in Southern China, Jiangsu Collaborative Innovation Center for Modern Crop Production, Nanjing Agricultural University, Nanjing, Jiangsu 210095 China

**Keywords:** Air temperature, Inorganic nitrogen concentration, Nitrogen metabolism enzyme activity, Soil bulk density, Water use efficiency

## Abstract

**Background:**

Alternate wetting and drying (AWD) irrigation improves water use efficiency (WUE) in rice, but its effectiveness in ratoon rice under varying climatic conditions remains unclear. This study is the first 3-year field experiment to assess the climate-dependent impacts of AWD irrigation on ratoon rice, particularly under extreme heat conditions.

**Methods:**

A 3-year field experiment (2020–2022) was conducted to examine AWD irrigation’s effects on soil properties, rice growth, yield, and WUE in a main-season–ratoon rice system. Four irrigation methods were tested: conventional irrigation (CK), AWD during the main-season rice filling period (T1), AWD during the ratoon rice filling period (T2), and AWD during both periods (T3). Weather data showed higher temperatures and lower rainfall in 2022 compared to 2020 and 2021.

**Results:**

AWD irrigation increased soil bulk density and total nitrogen, and and NO3− concentrations, while reducing NH4+ concentrations in both rice seasons. In 2020 and 2021, AWD irrigation positively increased root dry weight and activity, net photosynthetic rate, chlorophyll fluorescence, nitrogen metabolism enzyme activity, yield and WUE. However, the opposite was observed in 2022. Specifically, in 2020 and 2021, compared with CK, T1 and T3 increased the yield in the main season by 12.81% and 11.66%; T1, T2 and T3 increased the yield in the ratoon season by 12.93%, 7.66% and 24.10%; T1 and T3 increased the WUE in the main season by 14.90% and 14.07%; and T1, T2 and T3 increased the WUE in the ratoon season by 11.10%, 15.01% and 33.00%, respectively. In 2022, compared with CK, T1 and T3 reduced the yield in the main season by 12.04% and 12.60% and the yield in the ratoon season by 13.72% and 16.37%, and T1 and T3 reduced the WUE in the main season by 10.34% and 10.35% and the WUE in the ratoon season by 3.45% and 10.34%, respectively.

**Conclusions:**

AWD irrigation enhances yield and WUE under normal conditions but reduces them under extreme heat. This study highlights the need for adaptive irrigation strategies, integrating heat-tolerance measures to counteract the adverse effects of climate change on ratoon rice production.

## Introduction

Globally, rice (*Oryza sativa L.*) planting area covers 167 million hectares, with most cultivation occurring under continuous flood [1–4]. However, declining land and water resources present significant challenges to global food security [1–3, 5]. Rising air temperatures and frequent droughts further threaten rice productivity, particularly in regions prone to climate extremes [1–3, 6]. To address these challenges, the development of efficient and cost-effective water-saving irrigation techniques to enhance water use efficiency (WUE) and grain yield has become a primary concern for sustainable rice cultivation.

Alternate wetting and drying (AWD) irrigation––alternating between flooded and nonflooded soil conditions––is widely adopted for due to its dual benefits [1–3, 7, 8]. In rice cultivation, AWD contributes, fossil energy consumption, and production costs, while also mitigating environmental impacts and improving water use efficiency (WUE) [1–3, 9–11]. Despite these benefits, the impact of AWD on grain yield remains debated. Under AWD irrigation, rice yield may increase [1–3, 12, 13], remain stable [1–3, 14, 15] or decline [1–3, 16, 17]. Grain yield responses under AWD irrigation are influenced by climate, soil conditions, planting patterns and field management practices [1–3, 18]. The specific growth stage of application and the duration/intensity of the drying phase determine the effects of AWD irrigation on grain yield [1–3, 19, 20]. Generally, using safe AWD irrigation (where the field water level does not drop below 15 cm from the soil surface or field water potential is maintained ≥ -20 kPa) during noncritical growth stages of rice can maintain or even increase grain yield [14, 21]. Nevertheless, the interaction between AWD irrigation and extreme weather events, such as high air temperatures, remains poorly understood, particularly in systems with unique agronomic demands like ratoon rice.

Ratoon rice, a cultivation practice in which dormant buds on harvested stubble regenerate a second crop, provides a resource-efficient approach to enhancing cropping intensity and profitability [1–3]. Compared with single- or double-cropping systems, ratoon rice demands prolonged field management over two consecutive seasons, posing unique challenges for water and nutrient retention [22]. Recent research on AWD irrigation in ratoon rice has yielded inconsistent results. Zhang et al. [23] found that under normal field conditions, compared with conventional irrigation (CK), AWD irrigation increased the main-season and ratoon rice yields by 3.7% and 6.8%, respectively. Similarly, Ding et al. [24] reported that under normal field, AWD irrigation, compared with CK, increased the main-season and ratoon rice yields by 5.2% and 4.9%. Leavitt et al. [25] found under normal field, no significant difference in rice yield between CK and AWD irrigation. These inconsistencies underscore the need to examine how environmental factors, such as weather variability, influence AWD irrigation outcomes. Previous AWD irrigation studies on ratoon rice primarily examined normal climatic conditions, with minimal investigation into extreme weather scenarios [23–25]. High temperatures (> 35 °C) during grain filling impair rice physiology, pollination, nitrogen metabolism and assimilate partitioning [6]. However, whether AWD irrigation exacerbates or mitigates these effects under natural heatwaves remains unknown. This study addresses this gap through a 3-year field experiment comparing AWD irrigation performance under two normal-temperature years (2020–2021) and an extreme heat year (2022).

We hypothesised that AWD irrigation enhances yield under normal weather conditions but reduces yield under extreme high-temperature stress. To test this hypothesis, this study aimed to: (1) assess the effects of AWD irrigation on soil properties (bulk density and nitrogen dynamics), rice growth (root activity and photosynthesis) and yield components in a ratoon rice system; and (2) compare the efficacy of AWD irrigation under normal weather conditions versus extreme high-temperature stress.

## Materials and methods

### Site description

**Fig. 1:**
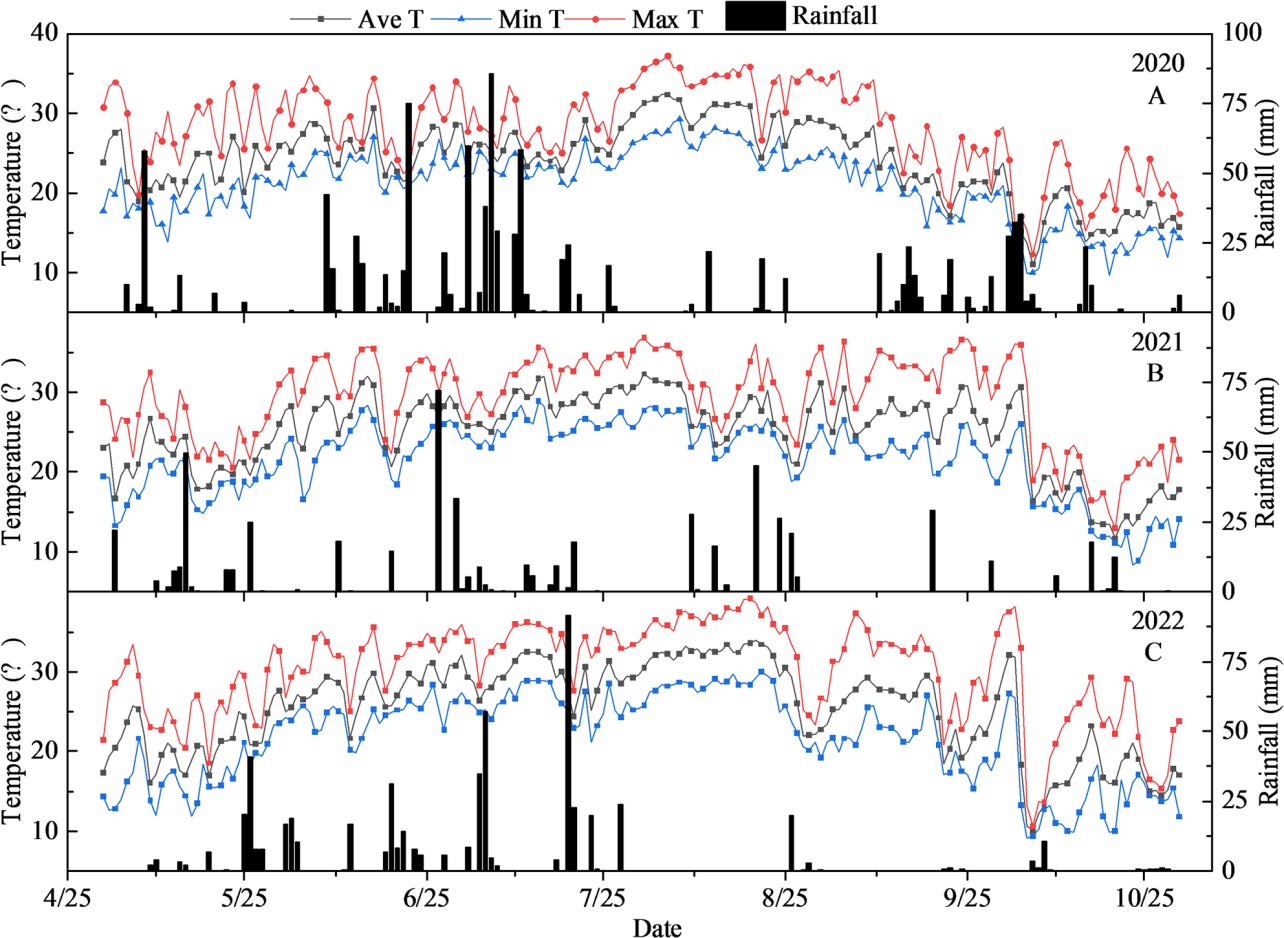
**A**-**C**. Weather conditions from main-season rice transplanting to ratoon rice harvest. Ave T, Min T and Max T represent average temperature, minimum temperature and maximum temperature, respectively

Field experiments were conducted at the experimental base in Yangtze University, Jingzhou City, Hubei Province, central China (112° 8′ 19″ E, 30° 21′ 19″ N). Experiments were conducted during the rice season (April–October) from 2020 to 2022. The region experiences subtropical monsoon humid climate. Daily air temperature and rainfall were recorded throughout the experimental period (Fig. 1A-C). The most notable climatic feature of 2022 was the coincidence of higher temperature and lower rainfall from main-season heading to ratoon maturity. Daily air temperature and rainfall during the experimental period are shown in Fig. 1A-C and summarised in Table 3. Based on the grain-filling period of main-season rice, 2020–2021 were treated as normal years, whereas 2022 was characterised by hotter and drier conditions.

In 2020, before fertilising and transplanting, soil samples were diagonally collected from the 0–20 cm soil layer to analyse the primary soil properties. The soil in the experimental area was classified as silty loam, composed of 28.2% sand, 57.1% silt and 14.7% clay. Soil pH was measured as 5.84 (soil/water, 1:2.5). Additionally, soil properties include organic matter content(OM) of 20.76 g kg^− 1^, total nitrogen (TN) of 1.43 g kg^− 1^, total phosphorus (TP) of 0.59 g kg^− 1^, total potassium (TK) of 2.57 g kg^− 1^, alkali-hydrolysed nitrogen(AN) of 82.57 mg kg^− 1^, available phosphorus(AP) of 46.75 mg kg^− 1^, available potassium(AK) of 114.54 mg kg^− 1^ and a bulk density (BD) of 1.27 g cm^− 3^. The rice cultivar used was YLiangyou911, with a total growth duration of 200–210 days across both the main and ratoon seasons. Before the start of the experiment, the rotation pattern at the site was main season–ratoon rice–winter fallow.

### Experimental design and management practices

**Fig. 2:**
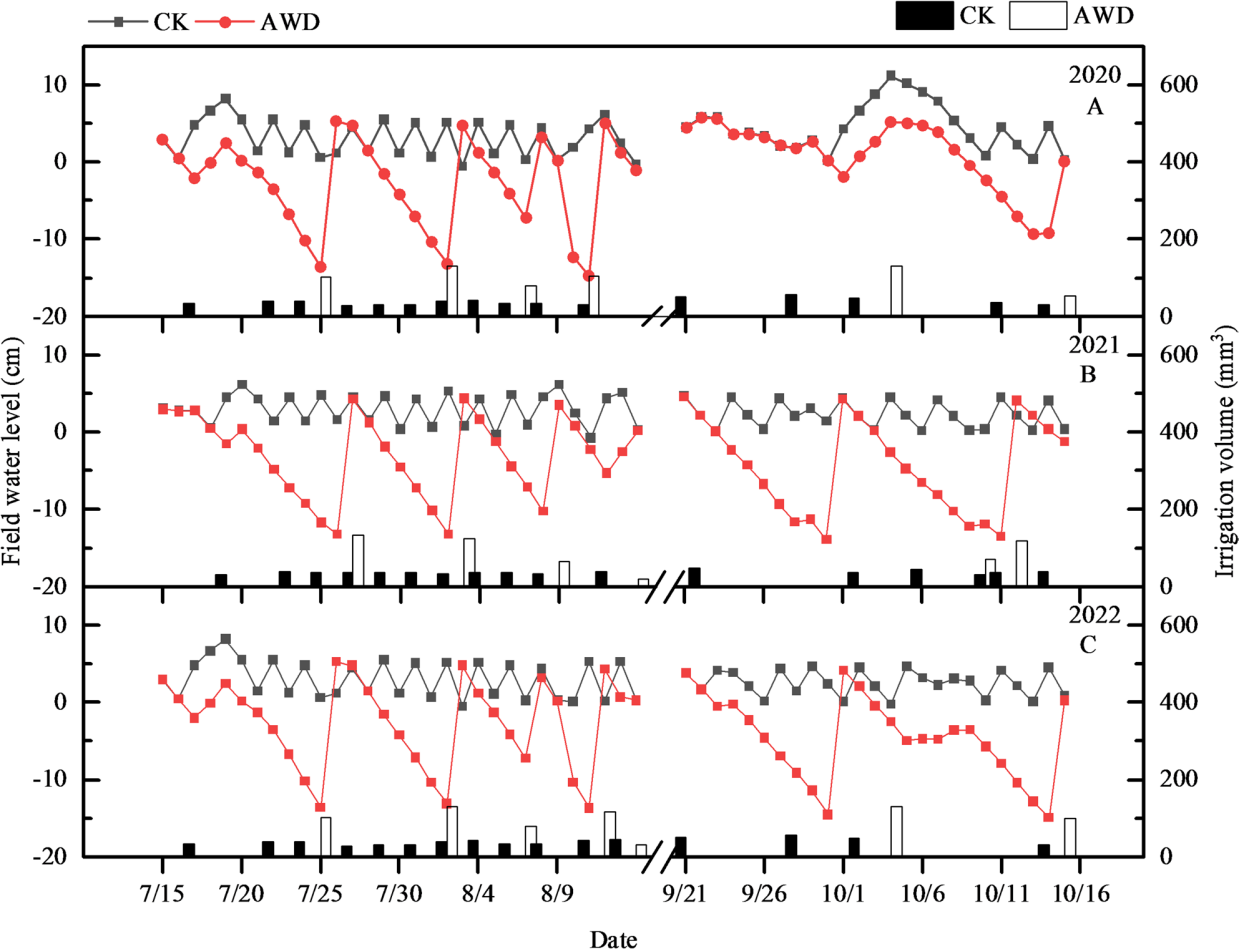
**A**-**C**. The changes of field water level and the daily irrigation volume. CK: conventional irrigation, AWD: alternate wetting and drying irrigation

This study employed a single-factor experimental design, with irrigation regime as the sole factor (four levels: CK, T1, T2, T3) and three replicates arranged in a randomised complete block design. The detailed irrigation operations for each treatment are shown in Table 1. Plots were separated by 30 cm-high bunds lined with plastic film inside and outside to prevent water seepage. Each plot had an independent irrigation and drainage system (equipped with a volumetric water meter and water level meter) to ensure strict water management and avoid cross-contamination of water between plots. The plot size was 60 m² (4 × 15 m). The drainage events in the CK reflect local farmers’ practices to reduce ineffective tillering and facilitate harvesting [26]. Field water depth was calculated using a water level metre (MYWT, Minyi, China). The irrigation volume for each plot was estimated using a volumetric water metre (LXS-15 F, Ningbo, China). Figure 2A-C illustrates field water level fluctuations during the dry and wet periods. Figure 2A-C illustrates that AWD treatments differed primarily in the timing and duration of the drying phase during grain filling, whereas CK maintained higher and more stable field water levels. This treatment contrast formed the basis for comparing physiological and yield responses under different climatic years. Table 2 presents the irrigation times, irrigation volume and total rainfall and water consumption. Irrigation water was drawn from an 8-m-deep underground well situated 2 m away from the experimental field.

**Table 1:** Detailed description of irrigation treatments

Treatment	Irrigation stage	Irrigation operation
CK (conventional irrigation)	Entire growth period	Three drainage events: (i) late tillering (5 cm below soil surface for 5 days), (ii) 7 days before main-season rice harvest, (iii) 7 days before ratoon rice harvest; water levels maintained at 2–5 cm otherwise
T1	Main-season rice filling period (end of flowering–maturity)	AWD irrigation: reirrigation initiated at 15 cm below soil surface, replenished to 5 cm standing water; other stages same as CK
T2	Ratoon rice filling period (end of flowering–maturity)	AWD irrigation: same thresholds as T1; other stages same as CK
T3	Main-season + ratoon rice filling periods	AWD irrigation: same thresholds as T1; other stages same as CK

**Table 2:** Irrigation times, irrigation volume and sum of rainfall and water consumption during other periods under different irrigation treatments in 2020, 2021 and 2022. Due to the lack of significant differences in field water level and irrigation volume between CK and T2 as well as T1 and T3 during the filling period of the main-season rice and CK and T1 as well as T2 and T3 during the filling period of the ratoon season rice, the four irrigation treatments were divided into CK and AWD based on their specific operations. CK and AWD represent conventional irrigation and alternate wetting and drying irrigation, respectively

Year	Treatment	Irrigation times		Irrigation volume		Sum of rainfall and water consumption during other periods	
		Main season	Ratoon season	Main season	Ratoon season	Main season	Ratoon season
				mm	mm	mm	mm
2020	CK	10	3	405 ± 17a	105 ± 4a	1096	388
	AWD	3	1	367 ± 8b	53 ± 2b		
2021	CK	11	7	400 ± 16a	373 ± 29a	976	285
	AWD	4	2	266 ± 13b	245 ± 12b		
2022	CK	11	7	431 ± 34a	396 ± 48a	1114	485
	AWD	4	2	248 ± 24b	220 ± 10b		

Rice seedlings were raised on 1 April and manually transplanted into the paddy field on 5 May from 2020 to 2022. Transplanting was performed with a hill spacing of 16 × 30 cm, placing two seedlings per hill. Main-season rice was harvested around 20 August, leaving a 30-cm-high stubble for ratoon rice growth. A 30-cm rice stubble is suitable for YLiangyou911 to be planted as ratoon rice in the experimental area [24]. Ratoon rice was harvested around 25 October. The rotation method of the experiment was main season–ratoon rice–winter fallow.

Each plot received 250 kg ha^− 1^ of nitrogen (N), 75 kg ha^− 1^ of phosphorus (P_2_O_5_) and 90 kg ha^− 1^ of potassium (K_2_O). These nutrients were supplied via urea (46% N), calcium superphosphate (12% P_2_O_5_) and potassium chloride (60% K_2_O). Prior to rice transplanting, base fertilisers were applied: 164 kg ha^− 1^ of urea, 625 kg ha^− 1^ of calcium superphosphate and 75 kg ha^− 1^ of potassium chloride. Topdressing fertilisers were administered at different growth stages. Specifically, urea was applied at rates of 98, 65, 108 and 108 kg ha^− 1^ during the tillering, heading and filling stages in the main-season rice and at the heading stage in the ratoon rice. Additionally, potassium chloride was administered at a rate of 75 kg ha^− 1^ during the filling stages in the main-season rice as a top-dressing fertiliser. To prevent yield losses and diseases, Pests, diseases, and weeds were managed in a timely manner following local agronomic recommendations to avoid confounding treatment effects.

### Sampling and measurements

#### Collection and determination of soil samples

During harvesting of the main-season and ratoon rice, the BD in the 0–20-cm soil layers was measured using a standard 100 cm^3^ cutting ring. Additionally, Rhizosphere soil was sampled 7 days before harvest to characterise soil nitrogen status immediately before maturity while minimising disturbance at final harvest. rhizosphere soil samples were diagonally collected from each plot at the 0–20-cm soil layer to analyse the concentration of TN, ammonia nitrogen (NH_4_^+^) and nitrate nitrogen (NO_3_^−^) following the method of Lu [27]. The TN concentration was determined via the Kjeldahl method. The NH_4_^+^ and NO_3_^−^ concentrations were measured using a continuous flow analyser (Alliance Fluura II, Huanyi, Shanghai, China).

#### Collection and determination of rice samples

During the middle filling stage of main-season and ratoon rice, five holes were selected at the centre of each plot to measure root dry weight (RDW), root activity (RA) and net photosynthetic rate (Pn) in rice plants. RDW and RA were assessed from samples taken from each hole. Each sample consisted of a soil–root mixture (25, 16 and 20 cm in length, width and depth), containing approximately 95% of the total root biomass [28]. Samples were carefully rinsed with tap water and then with distilled water. Approximately 1 g of fresh roots was extracted from the sample to determine RA using the α-naphthylamine oxidation method [29]. The remaining samples were dried at 70 °C until reaching a constant weight, then weighed. To determine Pn, the leaves were assessed using the LI-6400 portable photosynthesis measurement system. This measurement was taken on a sunny day between 9 and 11:30 am. The photosynthetic effective radiation above the canopy was maintained at 1500 µmol m^− 2^ s^− 1^, with the CO₂ concentration set to 400 µmol m⁻² s⁻¹.

During the middle filling stage of main-season and ratoon rice, the initial fluorescence (Fo), maximum fluorescence (Fm) and variable fluorescence (Fv) were measured using a chlorophyll fluorescence meter (Yaxin-1161G, China). The middle of the blade was clamped with a dark adaptation clip for 20 min, and then the fluorescence probe was drawn into the square groove of the dark adaptation clip. The measurement time was 1 s and the light intensity was 1500µmol·m-2·s^− 1^. Measurements were taken three times on both sides of the main vein.

During the grain-filling middle stage of the main-season and ratoon rice, five flag leaves were collected from each plot. These leaves were immediately frozen in liquid nitrogen and stored in an ultra-low-temperature refrigerator (-80 °C). This procedure aimed to assess the activities of nitrogen metabolic enzymes, such as nitrate reductase (NR), glutamine synthetase (GS) and glutamate synthase (GOGAT). The activities of NR, GS and GOGAT were measured using specific test kits [30]. NR activity was measured using a Nitrate Reductase Assay Kit (BC0080, Solarbio, Beijing, China). Meanwhile, 0.1 g of leaf samples was mixed with 1 ml of extraction solution and centrifuged at 4000 *g* for 10 min to determine NR. The supernatant was collected for analysis, and absorbance at 520 nm was recorded to calculate the NR activity. GS activity was determined using a Micro-Glutamine Synthetase Assay Kit (BC0915, Solarbio). For GS extraction, 0.1 g of the leaf sample was finely ground in liquid nitrogen, then extracted with 1 mL of extraction buffer. The mixture was centrifuged at 8000 *g* at 4 °C for 10 min, and the resulting supernatant was collected for activity measurement. The absorbance at 520 nm was used to calculate GS activity. GOGAT activity was determined using a Glutamate Synthase Assay Kit (BC0070, Solarbio). To extract GOGAT, 0.1 g of the leaf samples was mixed with 1 mL of the extraction buffer. The mixture was then centrifuged at 10,000 *g* at 4 °C for 10 min. The supernatant was collected, and the absorbance at 340 nm was recorded to determine GOGAT activity.

At the heading and ripening stages of the main-season and ratoon rice, five rice plants were collected from each plot. The sampled rice plants were divided into panicles, stems and leaves. These plant parts were deactivated at 105 °C for 30 min and dried at 70 °C until reaching a constant weight, then weighed. Based on the formulas provided by Jiang et al. [31], two indices of relevant dry matter were calculated: the redistribution amount of dry matter stored at preanthesis from vegetative organs to grain (RDM) and the accumulation amount of dry matter at postanthesis (ADM):

$$\begin{aligned} \mathrm{RDM} (\mathrm{t}\; \mathrm{ha} ^{-1}) &=\text{Dry matter of the stems and leaves at anthesis stage} \\&- \text{Dry matter of the stems and leaves at maturity stage} \end{aligned}$$

$$\mathrm{ADM} (\mathrm{t}\; \mathrm{ha} ^{-1}) = \text{Grain yield} - \mathrm{RDM}$$

The Kjeldahl method was used to determine the nitrogen concentration in grains and straw (stems and leaves) at maturity [32].

After the rice reaches maturity, three 1 m^2^ plots were randomly selected from each main plot to measure the effective panicles per unit area and yield. For seed testing, five representative hills of rice were collected from each plot. Additionally, total number of spikelets per panicle, the number of filled spikelets per panicle and the 1000-grain weight were recorded. The seed setting rate was calculated based on these measurements. The 1000-grain weight and yield were normalised to a grain moisture content of 14%. WUE was calculated by dividing the yield by the total water consumption, including rainfall and irrigation [33]. Note: 1 mm of water = 1 L m⁻² = 0.001 m³ m⁻², so the total water consumption (mm) was converted to m³ m⁻² by multiplying by 0.001. The corrected WUE formula is:

$$\mathrm{WUE} (\mathrm{kg}\; \mathrm{m} ^{-3}) = \frac{ \mathrm{Yield} \left(\mathrm{kg} \; \mathrm{m} ^{-2} \right)}{\mathrm{Rainfall} \left(\mathrm{mm} \right)\; \times \;\text{unit area} \left(\mathrm{m} ^{-2} \right) \;+\; \mathrm{irrigation} \left( \mathrm{mm}\right) \;\times\; \text{unit area} \left( \mathrm{m} ^{-2} \right) } \times {1000}$$

### Statistical analyses

Experimental data were collected from 2020 to 2022 and are presented as the mean ± standard error of three replicates. Data were analysed using SPSS 21.0 software. Data were analysed by two-way ANOVA with year (Y), irrigation treatment (T), and their interaction (Y × T) as fixed effects. When the ANOVA indicated significant treatment effects within a given year, means were separated using Duncan’s multiple range test at α = 0.05. Thus, p-values refer to ANOVA effects, whereas different letters indicate post hoc mean separation. Graphs were generated using the Origin Pro 2018 software.

## Result

### Weather conditions

Normal weather years (2020–2021) were defined as having average maximum temperatures < 35℃ during the grain-filling period of main-season rice, while the extreme heat year (2022) had an average maximum temperature of 35.8℃ (≥ 35℃, the critical threshold for rice heat stress) during this period [34]. As shown in Table 3, during the period from transplanting to heading in main-season rice, the average daily temperature was highest in 2021 (29.5 °C), followed by 2022 (26.1 °C) and 2020 (24.6 °C) and the average rainfall was highest in 2020 (9.1 mm), followed by 2022 (6.0 mm) and 2021 (4.2 mm). From heading to maturity of main-season rice, the average daily temperature was highest in 2022 (31.2 °C), followed by 2020 (29.5 °C) and 2021 (28.6 °C) and the average rainfall was highes in 2021 (3.0 mm), followed by 2020 (1.7 mm) and 2022 (1.4 mm). Notably, in 2022, the average maximum temperature from heading to maturity of main-season rice exceeded 35 °C (reaching 35.8 °C), which coincided precisely with the period of AWD irrigation implementation.The average temperature difference in the ratoon-season rice was < 2 °C; however, the average rainfall always highest in 2020 (4.6 mm), followed by 2021 (2.0 mm) and 2022 (0.7 mm). Overall, temperatures were higher and rainfall was lower in 2022 from the heading of the main-season rice to the maturity of the ratoon-season rice.

**Table 3:** Average temperature (Ave), minimum temperature (Min), maximum temperature (Max) and rainfall during different growth periods in 2020, 2021 and 2022

Year	Main season									Ratoon season								
	Transplant to heading Date 5/5–7/20				Heading to maturity Date 7/21 − 8/20				Maximum temperature during flowering period Date 7/15 − 7/25	Transplant to heading Date 8/21 − 9/25				Heading to maturity Date 9/25 − 10/25				Maximum temperature during flowering period Date 7/15 − 7/25
Ave	Min	Max	Rainfall	Ave	Min	Max	Rainfall		Ave	Min	Max	Rainfall	Ave	Min	Max	Rainfall		
2020	24.6	21.7	28.7	9.1	29.5	26.2	33.6	1.7	28.4	25.0	21.8	29.5	3.9	17.4	14.9	21.1	5.4	23.4
2021	29.5	25.5	32.3	4.2	28.6	25.8	32.8	3.0	33.3	27.0	23	32.2	2.3	19.5	16.4	24.0	1.6	33.3
2022	26.1	22.6	30.4	6.0	31.2	27.1	35.8	1.4	33.1	26.3	22.4	31.2	0.7	19.4	15	25.3	0.6	27.1

### Soil characteristics

**Fig. 3:**
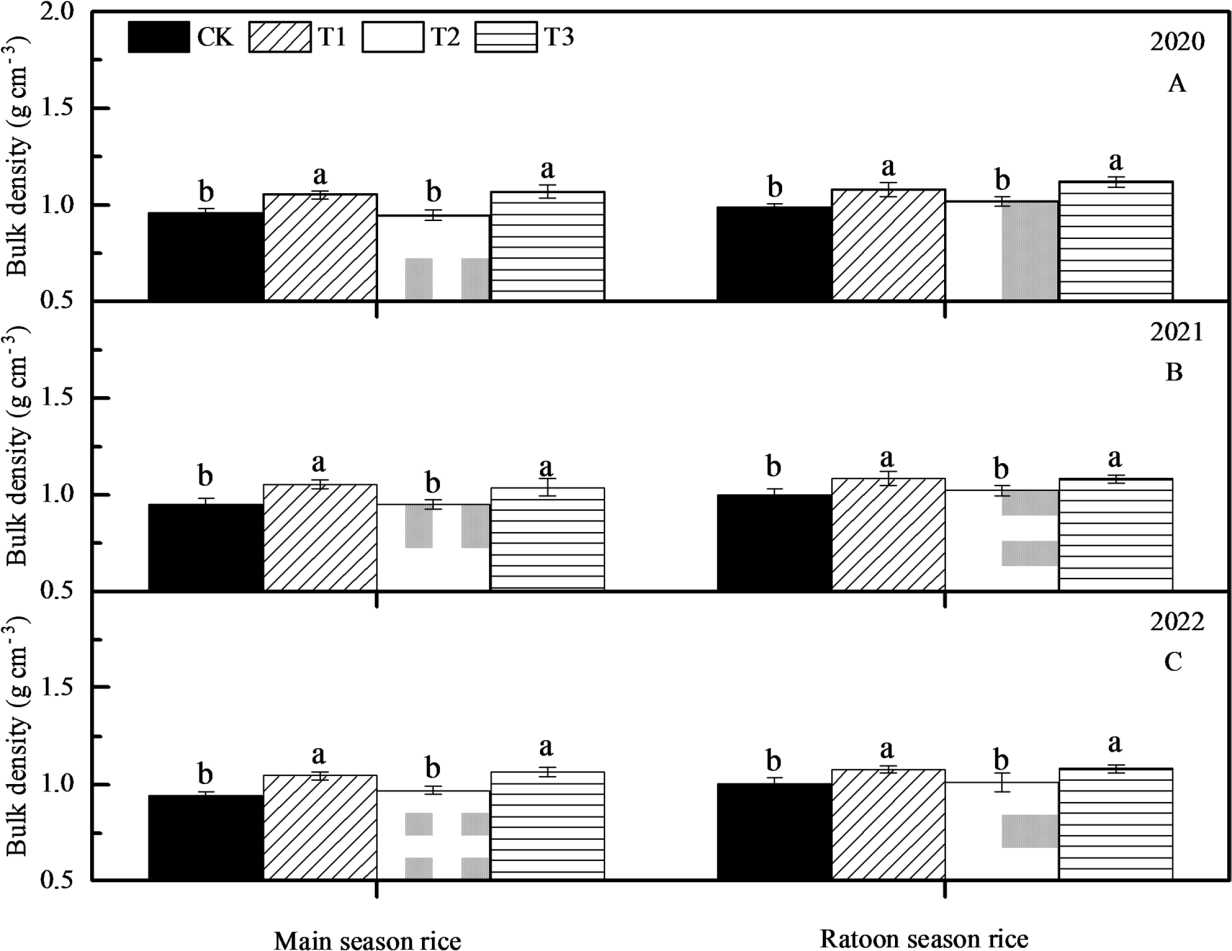
**A**-**C**. The impact of four irrigation treatments on soil bulk density. CK, T1, T2 and T3 represent conventional irrigation, AWD irrigation during the filling period of main-season rice, AWD irrigation during the filling period of ratoon rice, and AWD irrigation during the filling period of both main-season rice and ratoon rice, respectively. Values are means ± SE (*n* = 3). Different lowercase letters indicate significant differences among treatments within the same year, rice season (main or ratoon), and soil layer (Duncan’s test, α = 0.05)

**Fig. 4:**
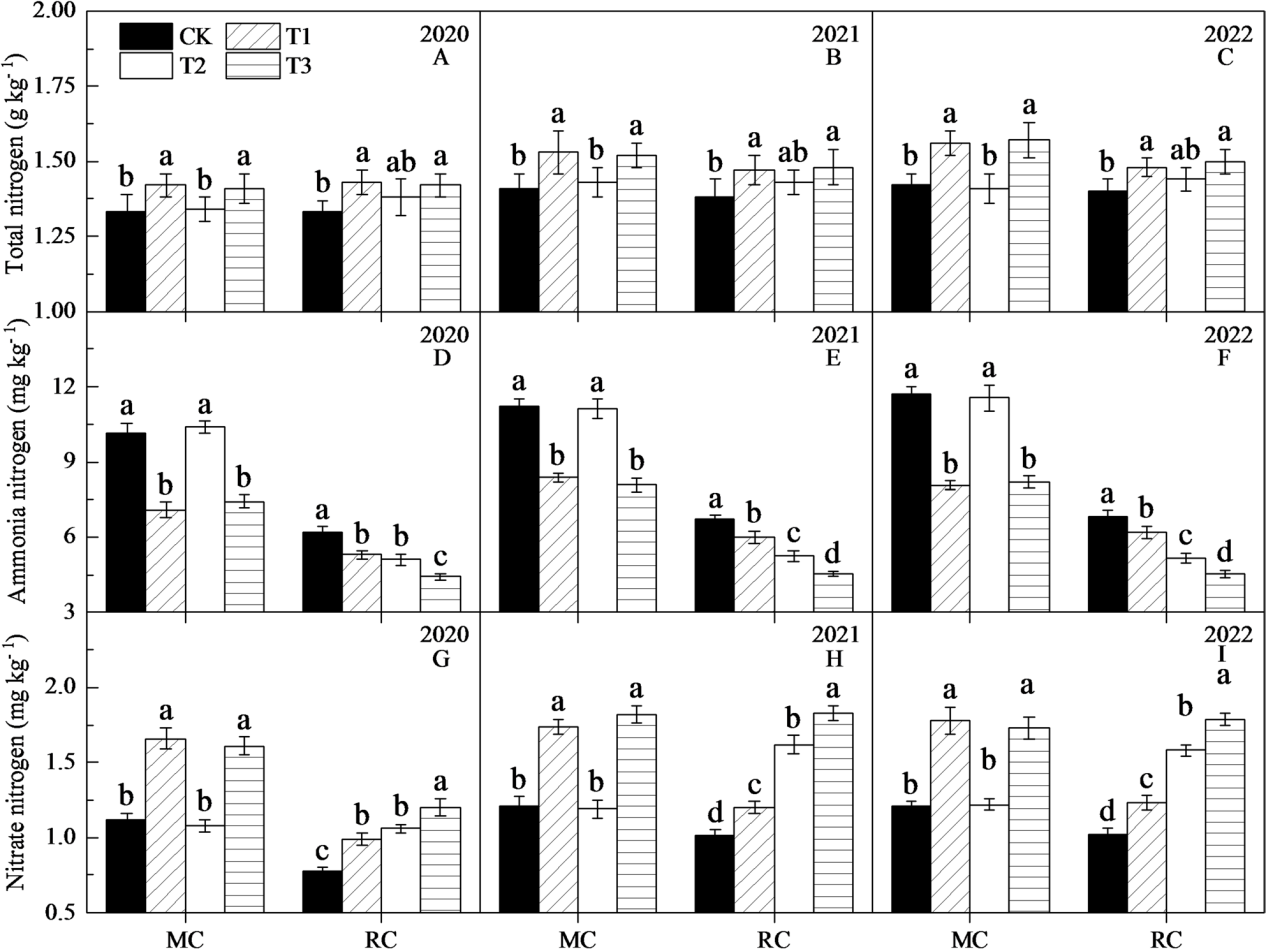
**A**-**I**. The impact of four irrigation treatments on the concentration of nitrogen. CK, T1, T2 and T3 represent conventional irrigation, AWD irrigation during the filling period of main-season rice, AWD irrigation during the filling period of ratoon rice, and AWD irrigation during the filling period of both main-season rice and ratoon rice, respectively. MC and RC represent main-season rice and ratoon season rice, respectively. Values are means ± SE (*n* = 3). Different lowercase letters indicate significant differences among treatments within the same year and rice season (MC or RC) according to Duncan’s multiple range test (α = 0.05)

Table 4 presents the results of all ANOVA tests. As shown in Fig. 3A-C, irrigation management during the main season of rice had a significant impact on the BD at the 0–20-cm soil layer. Compared with CK, T1 and T3 averagely increased the BD during harvesting of the main-season rice by 10.90% and averagely increased the BD during harvesting of the ratoon rice by 9.06%. Year had a significant effect on the TN concentrations in both main-season and ratoon rice, as well as the NO_3_^−^ concentration in ratoon rice (Fig. 4A-I). Specifically, the concentrations of these nitrogen fractions were significantly lower in 2020 than in 2021 and 2022. The lower nitrogen concentrations in 2020 compared to 2021 and 2022 may be attributed to the heavy rainfall on 8 May 2020, which likely caused nitrogen loss via runoff (Table 3). Heavy rainfall likely caused nitrogen loss from the applied base fertiliser owing to runoff in the field. Nevertheless, irrigation management had a significant impact on the concentrations of TN, NH_4_^+^ and NO_3_^−^.

**Table 4:** Analysis of year (Y), treatment (T), and the interaction of Y × T on bulk density (BD), concentration of total nitrogen (TN), ammonia nitrogen (NH4+) and nitrate nitrogen (NO3−), root dry weight (RDW), root activity (RA), net photosynthetic rate (Pn), minimum fluorescence (F0), maximal photochemical efficiency (Fv/Fm), actual photo chemical efficiency (Fv’/Fm’), electron transfer rate (ETR), the activities of nitrate reductase (NR), glutamine synthetase (GS) and glutamate synthase (GOGAT), nitrogen concentration in straw (NS) and in grain (NG), panicles per unit area (Panicles), total number of spikelets per panicle, the number of filled spikelets per panicle (Spikelets panicle), grain filling rate, 1000-grain weight, redistribution amount of dry matter stored in pre-anthesis from vegetative organs to grain (RDM), transfer amount of dry matter accumulated in postanthesis to grain (ADM), yield and water use efficiency (WUE) in 2020, 2021 and 2022

		BD (0-20)	BD (20-40)	TN	NH_4_^+^	NO_3_^−^	RDW	RA	Pn	F0
Main season	Y	ns	ns	**	ns	ns	*	ns	ns	ns
	T	**	ns	**	**	**	**	**	**	ns
	Y *T	ns	ns	ns	ns	ns	ns	ns	ns	ns
Ratoon season	Y	ns	ns	*	ns	**	**	**	**	**
	T	**	ns	**	**	**	ns	ns	ns	ns
	Y *T	ns	ns	ns	*	**	**	**	**	ns

		Fv/Fm	Fv’/Fm’	ETR	NR	GS	GOGAT	NS	NG	
Main season	Y	*	ns	ns	ns	ns	ns	**	**	
	T	*	ns	ns	**	**	**	*	ns	
	Y *T	**	**	**	ns	ns	ns	ns	ns	
Ratoon season	Y	**	**	**	**	**	**	ns	ns	
	T	ns	ns	ns	ns	ns	ns	**	**	
	Y *T	ns	**	**	**	**	**	ns	**	

		Panicles	Spikelets panicle	Grain filling rate	1000-grain weight	RDM	ADM	Yield	WUE	
Main season	Y	*	ns	**	ns	ns	ns	ns	**	
	T	ns	ns	ns	ns	ns	ns	ns	ns	
	Y *T	ns	ns	ns	**	**	ns	**	**	
Ratoon season	Y	**	**	**	**	**	**	**	**	
	T	ns	ns	ns	ns	ns	ns	ns	ns	
	Y *T	ns	**	ns	**	**	ns	**	**	

Specifically, compared with CK, T1 and T3 averagely increased the TN and NO_3_^−^ concentrations in main-season rice by 8.16% and 45.63%, respectively, while reducing the NH_4_^+^ concentration by 28.60%; for ratoon rice, T1, T2 and T3 increased the TN concentration by 6.73%, 3.41% and 7.14%, decreased the NH_4_^+^ concentration by 11.37%, 21.23% and 31.70%, and increased the NO_3_^−^ concentration by 21.31%, 51.25% and 71.13%, respectively. Additionally, the the interaction of Y × T significantly impacted the concentration of NH_4_^+^ and NO_3_^−^ in the ratoon rice.

### Root function and photosynthetic characteristics

**Fig. 5:**
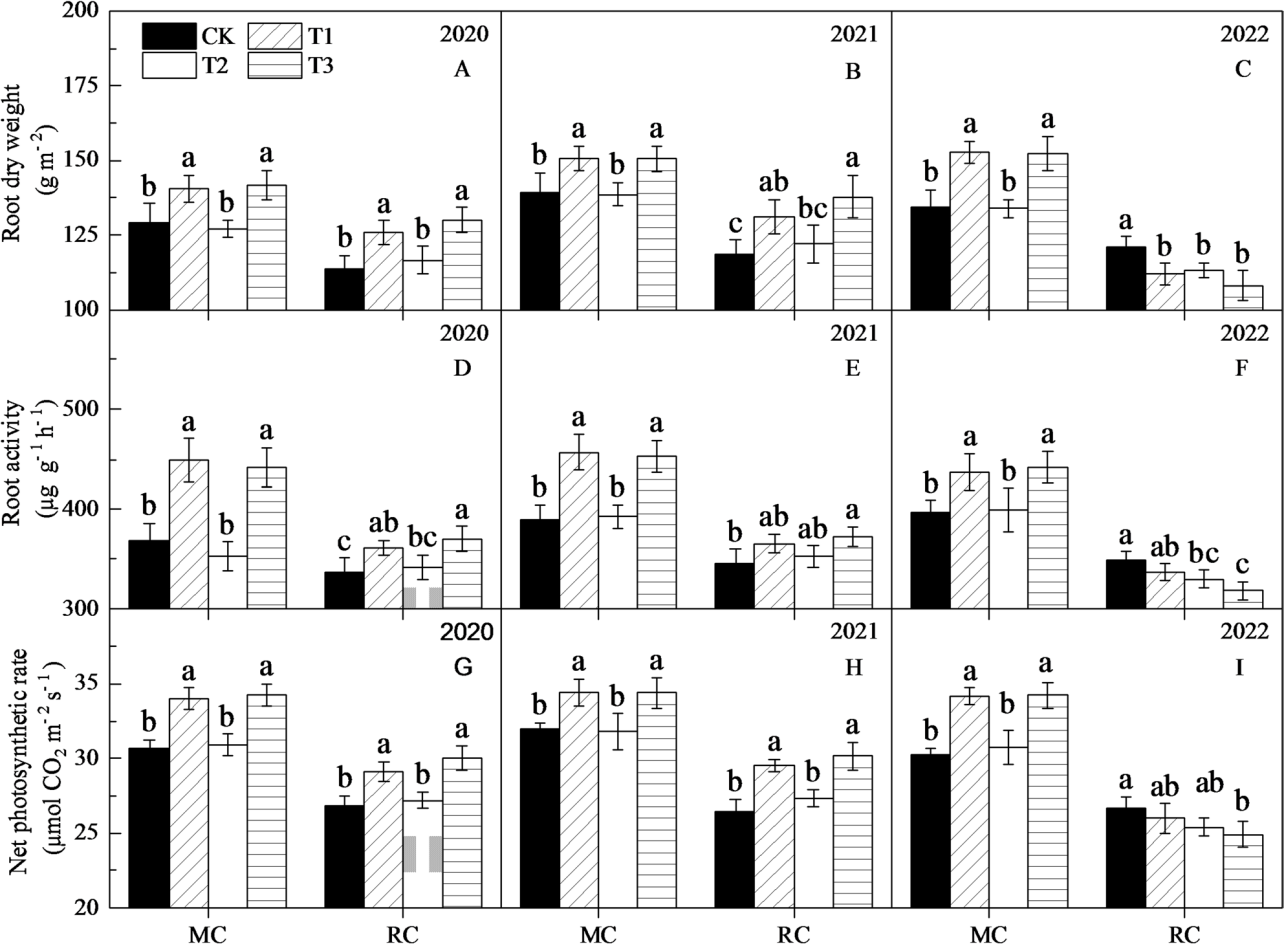
**A**-**I**. Effect of irrigation treatments on the root dry weight, root activity and net photosynthetic rate. CK, T1, T2 and T3 represent conventional irrigation, AWD irrigation during the filling period of main-season rice, AWD irrigation during the filling period of ratoon rice, and AWD irrigation during the filling period of both main-season rice and ratoon rice, respectively. MC and RC represent main-season rice and ratoon season rice, respectively. Values are means ± SE (*n* = 3). Different lowercase letters indicate significant differences among treatments within the same year and rice season (MC or RC) according to Duncan’s multiple range test (α = 0.05)

As shown in Fig. 5A-I, year had a significant effect on the RDW of both main-season and ratoon rice, as well as the RA and Pn of ratoon rice. Specifically, in main-season rice, the RDW in 2020 was significantly lower than that in 2021 and 2022; for ratoon rice, the magnitudes of RDW, RA and Pn followed a consistent interannual order of 2021 > 2020 > 2022. These differences in year are primarily attributed to weather conditions. Irrigation management had a significant impact on the RDW, RA and Pn of main-season rice. Specifically, compared with CK, T1 and T3 averagely increased the RDW, RA and Pn of main-season rice by 10.33%, 16.18% and 10.53%, respectively. For ratoon rice, the response to irrigation management was highly dependent on climatic conditions: in 2020 and 2021 (normal temperature years), compared with CK, T1, T2 and T3 averagely increased RDW by 10.55%, 2.66% and 15.28%, RA by 6.56%, 1.84% and 8.85%, and Pn by 10.08%, 2.34% and 12.92%, respectively; however, in 2022 (extreme heat year), the trends reversed—compared with CK, T1, T2 and T3 significantly decreased RDW by 20.38%, 21.42% and 24.61%, RA by 8.46%, 10.45% and 14.61%, and Pn by 13.10%, 17.34% and 20.36%, respectively.

**Fig. 6:**
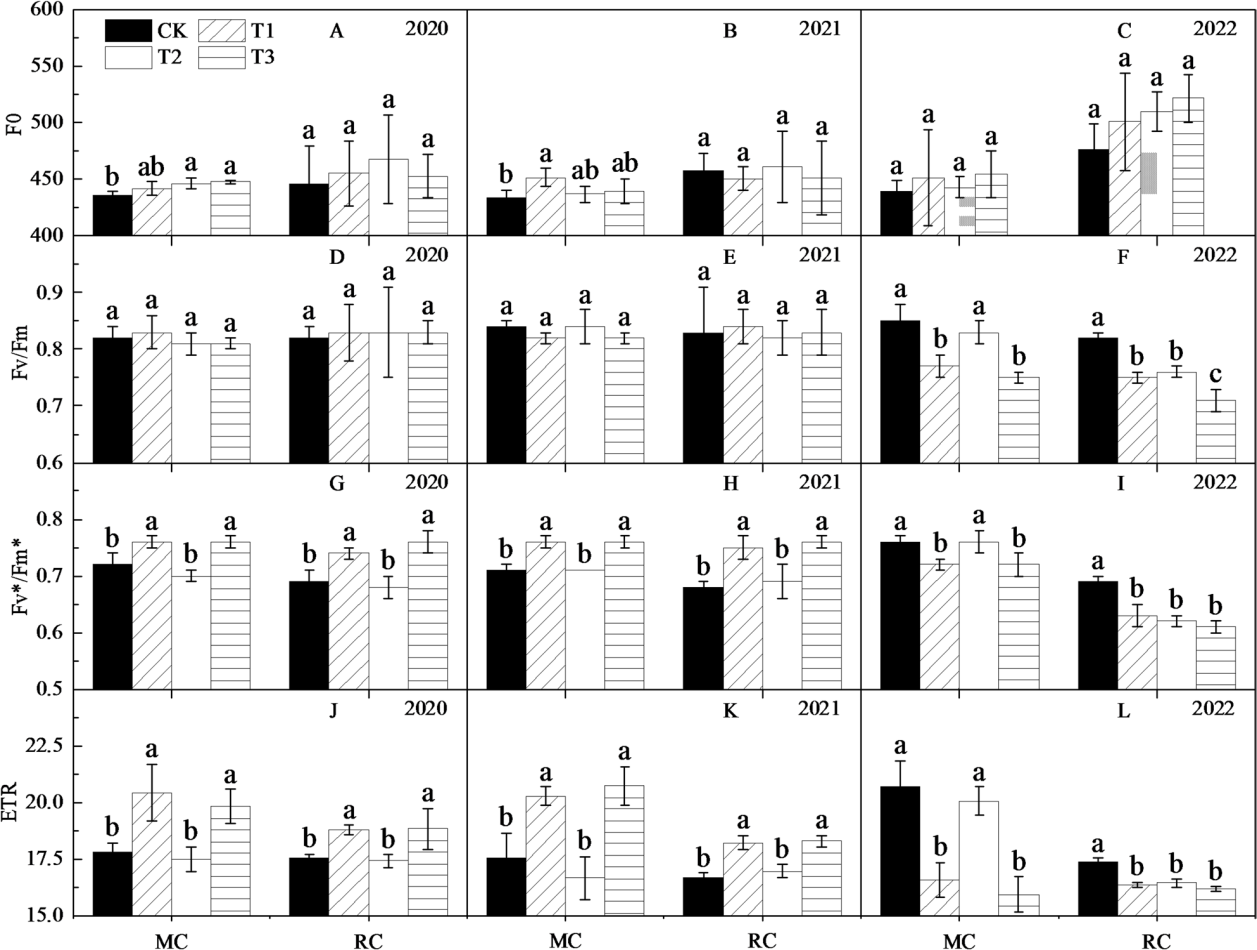
**A**-**L**. Effect of irrigation treatments on the F0, Fv/Fm, Fv’/Fm’ and ETR. CK, T1, T2 and T3 represent conventional irrigation, AWD irrigation during the filling period of main-season rice, AWD irrigation during the filling period of ratoon rice, and AWD irrigation during the filling period of both main-season rice and ratoon rice, respectively. MC and RC represent main-season rice and ratoon season rice, respectively. Values are means ± SE (*n* = 3). Different lowercase letters indicate significant differences among treatments within the same year and rice season (MC or RC) according to Duncan’s multiple range test (α = 0.05)

As shown in Fig. 6A-L, there were significant differences in Fv/Fm of main-season rice and F0, Fv/Fm, Fv’/Fm’ and ETR of ratooning rice in different years. Specifically, in main-season rice, Fv/Fm in 2022 was significantly lower than that in 2021 and 2020. In the ratoon rice, the Fo in 2022 was significantly higher than that in 2020 and 2021, the Fv/Fm, Fv’/Fm’ and ETR in 2022 were significantly lower than that in 2020 and 2021. The interaction of Y × T significantly influenced Fv/Fm, Fv’/Fm’ and ETR of main rice and Fv’/Fm’ and ETR of ratoon rice. Specifically, in the main-season rice, in 2020 and 2021, compared with CK, T1 and T3 averagely increased Fv/Fm, Fv’/Fm’ and ETR by 0.81%, 6.55% and 14.85%, respectively, in 2022, compared with CK, T1 and T3 averagely decreased Fv/Fm, Fv’/Fm’ and ETR by 10.44%, 5.29% and 21.54%, respectively. In the ratoon rice, in 2020 and 2021, compared with CK, T1, T2 and T3 had average increases of 8.51%, 0.24% and 10.71% under Fv’/Fm’ and 8.10%, 0.49%, 8.43% under ETR; In 2022, the results reversed. Compared with CK, T1, T2 and T3 showed declines of 8.70%, 9.67% and 12.07% under Fv’/Fm’; 57 7%, 53.0% ,67.7% under ETR, respectively.

### Nitrogen metabolism, enzyme activity and nitrogen concentration in rice

**Fig. 7:**
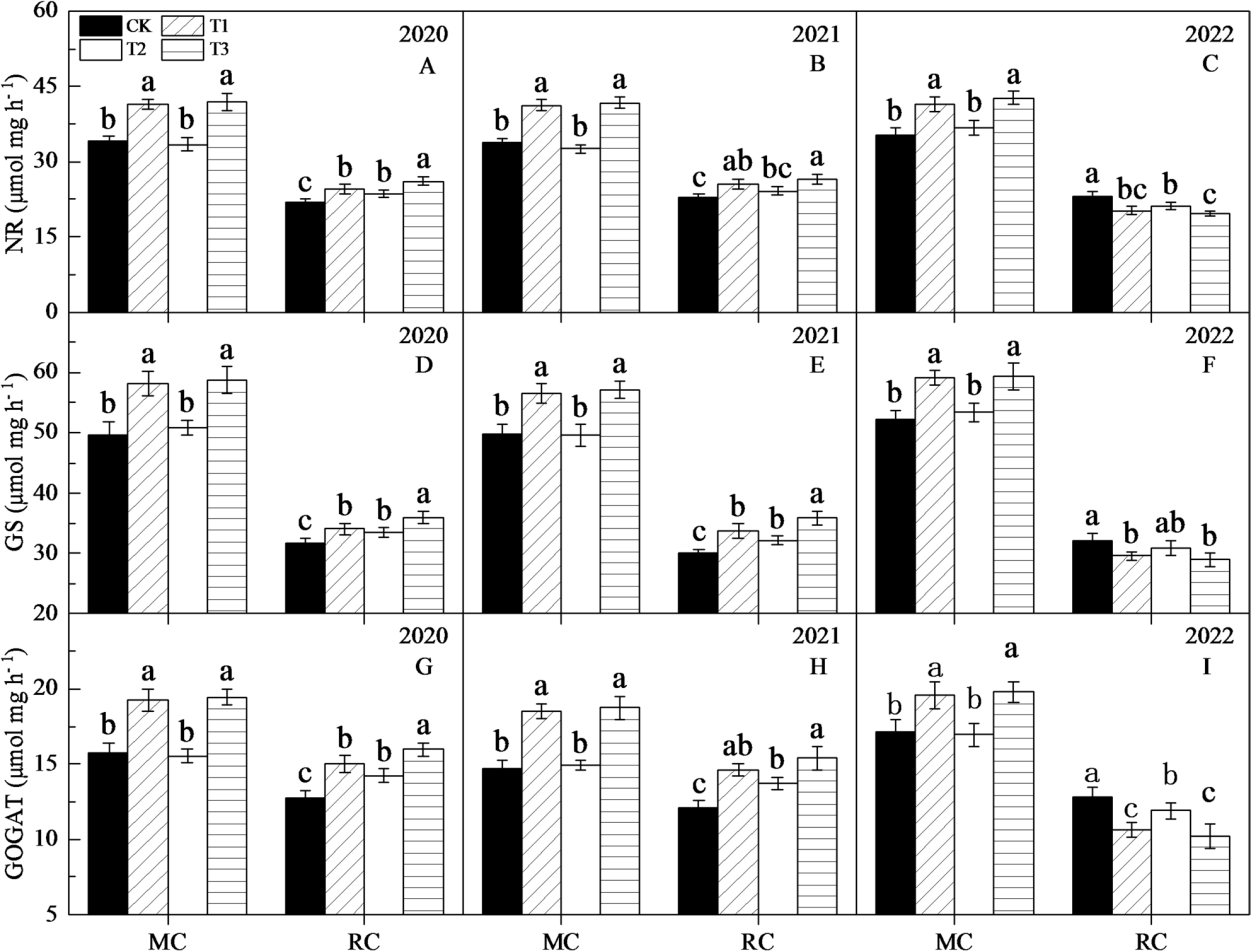
**A**-**I**. Effect of irrigation treatments on the activities of NR, GS and GOGAT. CK, T1, T2 and T3 represent conventional irrigation, AWD irrigation during the filling period of main-season rice, AWD irrigation during the filling period of ratoon rice, and AWD irrigation during the filling period of both main-season rice and ratoon rice, respectively. MC and RC represent main-season rice and ratoon season rice, respectively. Values are means ± SE (*n* = 3). Different lowercase letters indicate significant differences among treatments within the same year and rice season (MC or RC) according to Duncan’s multiple range test (α = 0.05)

As shown in Fig. 7A-I, there were significant differences in the activities of NR, GS and GOGAT of ratooning rice in different years. Specifically, the activities of NR, GS and GOGAT in 2022 was significantly lower than that in 2020 and 2021. Irrigation management had a significant impact on the activities of NR, GS and GOGAT in main-season rice. Specifically, compared with CK, T1 and T3 averagely increased the NR, GS and GOGAT activities of main-season rice by 21.64%, 15.07% and 21.14%, respectively. Additionally, the interaction of Y × T significantly influenced the activities of these three enzymes in ratoon rice. In 2020 and 2021, compared with CK, T1, T2 and T3 had average increases of 11.77%, 6.62% and 17.63% under the activities of NR; 9.66%, 6.09% and 16.21% under the activities of GS; 19.26%, 12.53% and 26.35% under the activities of GOGAT; In 2022, the results reversed. Compared with CK, T1, T2 and T3 showed declines of 12.83%, 14.45% and 18.45% under the activities of NR; 11.00%, 12.97% and 18.16% under the activities of GS; 14.22%, 17.37% and 20.03% under the activities of GOGAT, respectively.

**Fig. 8:**
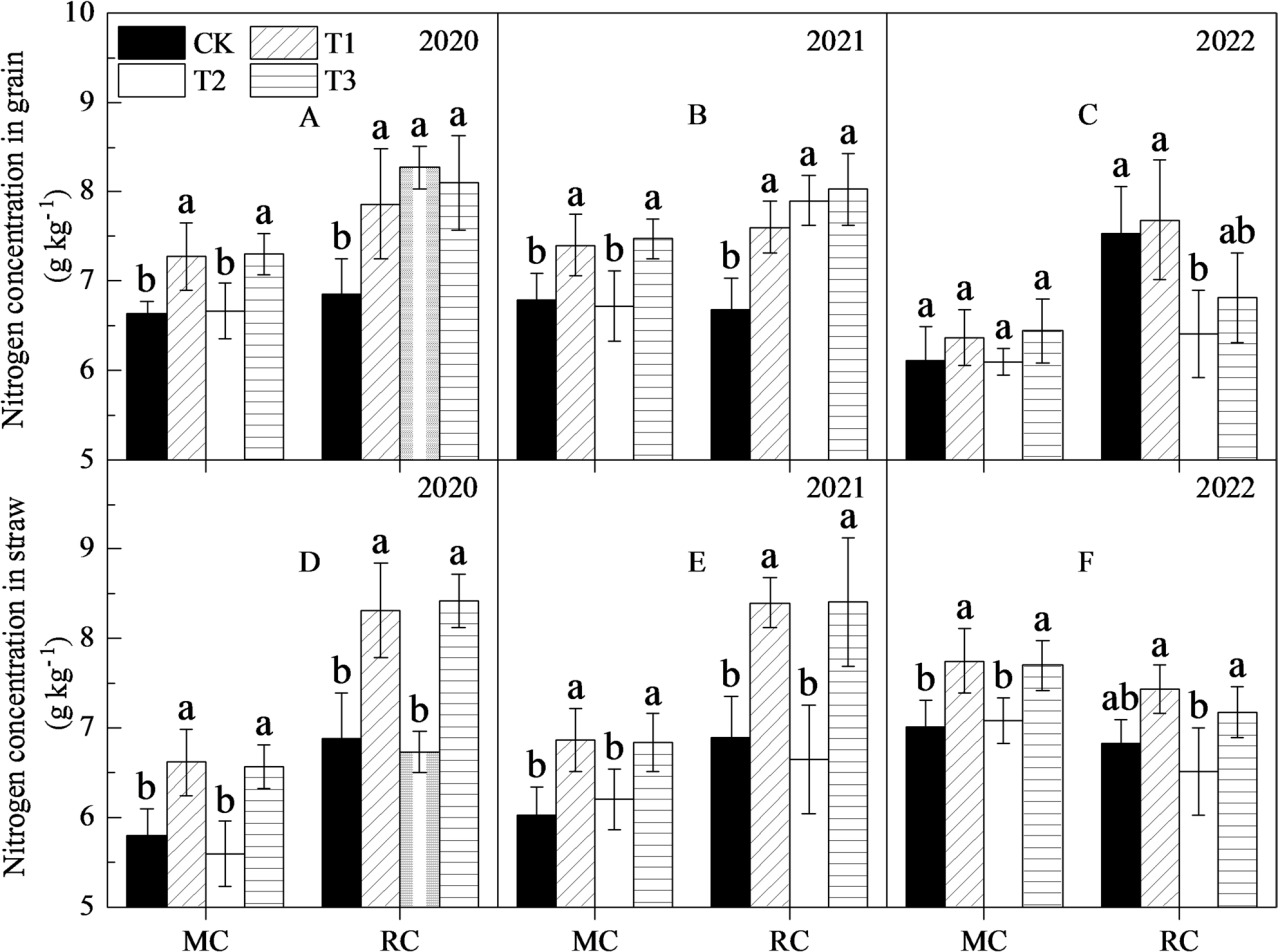
**A**-**F**. The impact of four irrigation treatments on the nitrogen concentration in grain and straw. CK, T1, T2 and T3 represent conventional irrigation, AWD irrigation during the filling period of main-season rice, AWD irrigation during the filling period of ratoon rice, and AWD irrigation during the filling period of both main-season rice and ratoon rice, respectively. MC and RC represent main-season rice and ratoon season rice, respectively. Values are means ± SE (*n* = 3). Different lowercase letters indicate significant differences among treatments within the same year and rice season (MC or RC) according to Duncan’s multiple range test (α = 0.05)

As shown in Fig. 8A-F, significant differences were observed in nitrogen concentration in the main-season straw and grains over different years. The nitrogen concentration in grains was higher in 2022 than those in 2020 and 2021, whereas the nitrogen concentration in straw exhibited the opposite trend. The reason may be that the high temperature during the filling period disrupted the transport of nitrogen from stems and leaves to the panicles, allowing more nitrogen to remain in the straw rather than in the grains. Moreover, irrigation management played a significant role in determining the nitrogen concentration in the straw of main-season rice and the nitrogen concentration in the straw and grains of ratoon rice. In comparison to CK, T1 and T3 showed an average increase of 12.45% in the nitrogen concentration of the straw of the main-season rice. Additionally, compared with CK, the nitrogen concentration in the straw of ratoon rice increased by 17.29% and 16.61% under T1 and T3, respectively, and decreased by 3.40% under T2; the nitrogen concentration in the grain of ratoon rice increased by 9.88%, 7.22% and 8.93% under T1, T2 and T3. The interaction of Y × T had a significant impact on the nitrogen concentration in the ratoon rice grains. In 2020 and 2021, compared with CK, the nitrogen concentration in the grains of ratoon rice increased by 14.26%, 19.50% and 19.23% under T1, T2 and T3, respectively. However, in 2022, the nitrogen concentration in the grains of ratoon rice decreased by 14.87% and 9.56% under the T2 and T3 treatments, respectively.

### Yield component factors and yield formation

As shown in Table 5, significant differences were observed in the effective panicles per unit area and grain-filling rate of the main-season rice and the four yield components of ratoon rice over different years. In main-season rice, the effective panicles per unit area followed the order of 2021 > 2022 > 2020, whereas the grain-filling rate followed the order of 2020 > 2021 > 2022. In addition, all four yield components of ratoon rice were lowest in 2022. The interaction of Y × T had a significant impact on the 1000-grain weight of the main-season rice, the number of spikelets per panicle and the 1000-grain weight of ratoon rice. In 2020 and 2021, compared with CK, T1 and T3 exhibited increases of 5.54% and 6.22% in the 1000-grain weight of the main-season rice and increases of 12.05% and 12.13% in the number of spikelets per panicle of ratoon rice, and T2 and T3 exhibited increases of 6.86% and 6.38% in the 1000-grain weight of ratoon rice, respectively. However, in 2022, compared with CK, T1 and T3 exhibited decreases of 7.43% and 7.55% in the 1000-grain weight of the main-season rice and decreases of 7.48% and 7.05% in the number of spikelets per panicle of the main-season rice, and T1 and T2 exhibited decreases of 3.94% and 4.59% in the 1000-grain weight of ratoon rice, respectively.

**Table 5:** The impact of four irrigation treatments on the yield components of main-season rice and ratoon rice. CK, T1, T2 and T3 represent conventional irrigation, AWD irrigation during the filling period of main-season rice, AWD irrigation during the filling period of ratoon rice, and AWD irrigation during the filling period of both main-season rice and ratoon rice, respectively. Values are means ± SE (*n* = 3). Different lowercase letters indicate significant differences among treatments within the same year according to Duncan’s multiple range test (α = 0.05)

Year	Treatment	Main season				Ratoon season			
		Panicles	Spikelets panicle^− 1^	Grain filling rate	1000-grain weight	Panicles	Spikelets panicle^− 1^	Grain filling rate	1000-grain weight
		No m^− 2^		%	g	No m^− 2^		%	g
2020	CK	222.74 ± 13.17a	216.11 ± 11.76a	79.57 ± 1.65a	23.39 ± 0.77b	239.84 ± 6.43a	101.33 ± 4.17b	53.15 ± 1.12a	22.02 ± 0.48b
	T1	225.79 ± 8.20a	220.00 ± 11.36a	82.06 ± 1.48a	24.71 ± 0.70a	251.94 ± 9.85a	113.48 ± 6.34a	51.76 ± 1.67a	21.89 ± 0.85b
	T2	224.00 ± 7.09a	213.00 ± 9.81a	80.07 ± 2.46a	23.27 ± 0.47b	241.41 ± 8.51a	102.97 ± 5.67b	52.01 ± 1.89a	23.57 ± 0.68a
	T3	223.52 ± 7.39a	220.22 ± 6.66a	82.11 ± 3.65a	24.96 ± 0.58a	252.23 ± 5.53a	113.91 ± 5.56a	53.01 ± 2.45a	23.48 ± 0.25a
2021	CK	232.38 ± 8.37a	218.37 ± 10.49a	77.45 ± 3.23a	23.53 ± 0.45b	246.61 ± 8.30a	99.51 ± 3.50b	52.18 ± 1.68a	21.87 ± 0.64b
	T1	236.08 ± 7.44a	220.33 ± 8.05a	79.89 ± 1.76a	24.81 ± 0.57a	255.73 ± 12.74a	111.57 ± 6.34a	50.42 ± 1.20a	21.83 ± 0.45b
	T2	232.37 ± 6.00a	218.59 ± 9.31a	77.80 ± 2.89a	23.24 ± 0.55b	251.08 ± 6.25a	100.25 ± 5.59b	51.41 ± 1.370a	23.33 ± 0.45a
	T3	234.41 ± 13.56a	220.79 ± 9.05a	79.73 ± 1.76a	24.88 ± 0.64a	254.25 ± 4.46a	111.29 ± 3.24a	52.14 ± 2.20a	23.21 ± 0.71a
2022	CK	228.70 ± 7.56a	223.46 ± 6.03a	77.21 ± 2.37a	25.17 ± 0.88a	241.91 ± 8.24a	97.89 ± 4.70a	48.92 ± 1.26b	21.59 ± 0.68a
	T1	232.74 ± 11.91a	219.39 ± 10.84a	73.82 ± 1.84a	23.30 ± 0.49b	233.15 ± 6.19a	90.57 ± 2.64b	48.15 ± 1.93b	21.28 ± 0.63a
	T2	230.41 ± 7.39a	223.30 ± 12.07a	77.30 ± 2.25a	24.82 ± 0.53a	243.21 ± 11.14a	97.38 ± 2.12a	49.25 ± 2.45b	20.74 ± 0.72a
	T3	230.00 ± 8.79a	218.20 ± 7.54a	74.53 ± 2.99a	23.27 ± 0.51b	234.56 ± 8.61a	90.99 ± 1.46b	47.55 ± 1.12b	20.60 ± 0.61a

**Fig. 9:**
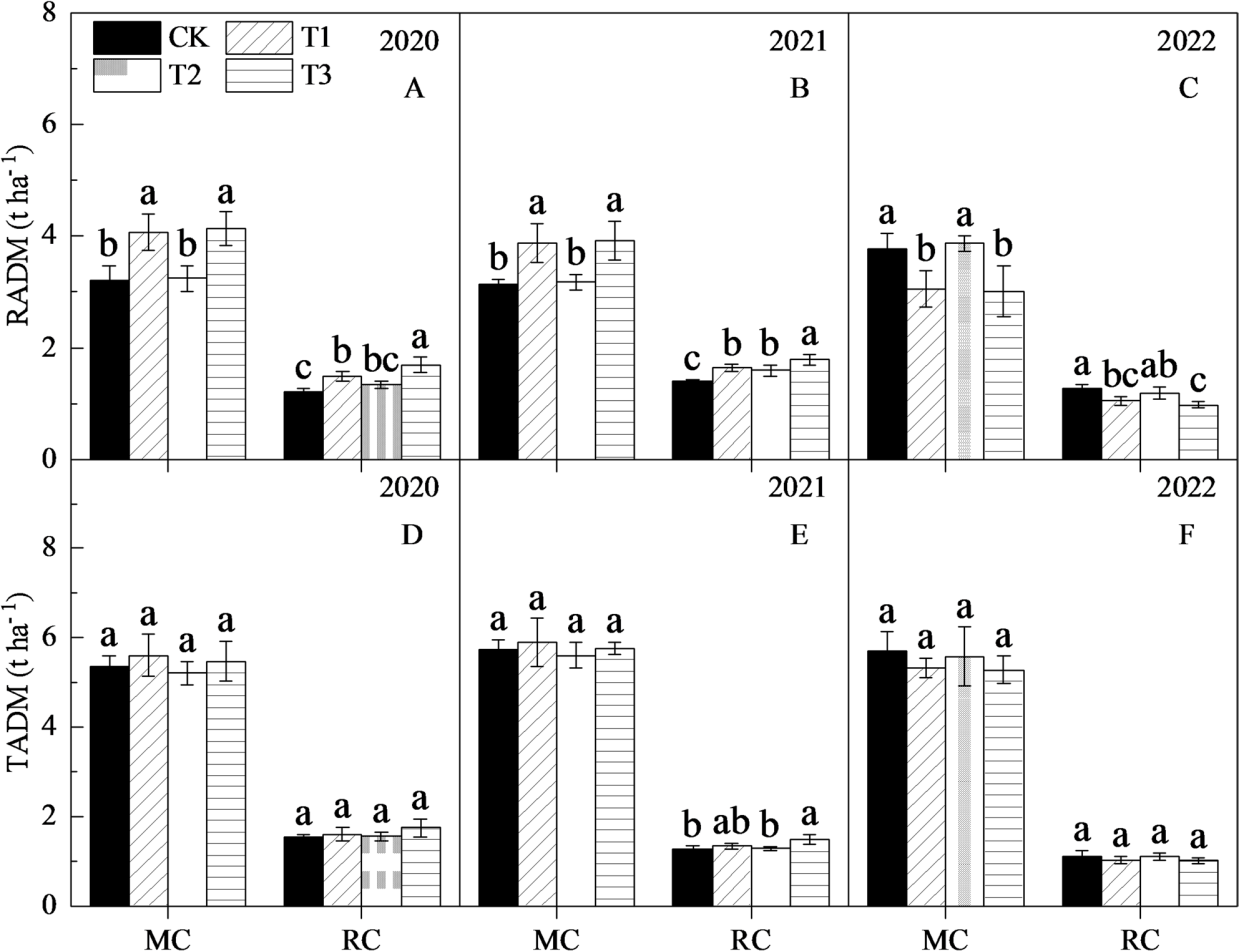
**A**-**F**. The impact of four irrigation treatments on the RADM and TADM. CK, T1, T2 and T3 represent conventional irrigation, AWD irrigation during the filling period of main-season rice, AWD irrigation during the filling period of ratoon rice, and AWD irrigation during the filling period of both main-season rice and ratoon rice, respectively. MC and RC represent main-season rice and ratoon season rice, respectively. Values are means ± SE (*n* = 3). Different lowercase letters indicate significant differences among treatments within the same year and rice season (MC or RC) according to Duncan’s multiple range test (α = 0.05)

As shown in Fig. 9A-F, significant differences were observed in the RDM and ADM of ratoon rice over different years. The RDM and ADM were lower in 2022 than those in 2020 and 2021. The interaction of Y × T had a significant impact on RDM both in main-season rice and ratoon rice. In 2020 and 2021, compared with CK, the RDM of the main-season rice increased by 25.57% and 27.15% under T1 and T3, and the RDM of the ratoon rice increased by 20.52%, 12.62% and 34.36% under T1, T2 and T3, respectively. However, in 2022, compared with CK, RDM of the main-season rice decreased by 19.10% and 20.16% under T1 and T3, and the RDM of the ratoon rice decreased by 17.76%, 7.32% and 22.98% under T1, T2 and T3, respectively.

### Yield and WUE

**Fig. 10:**
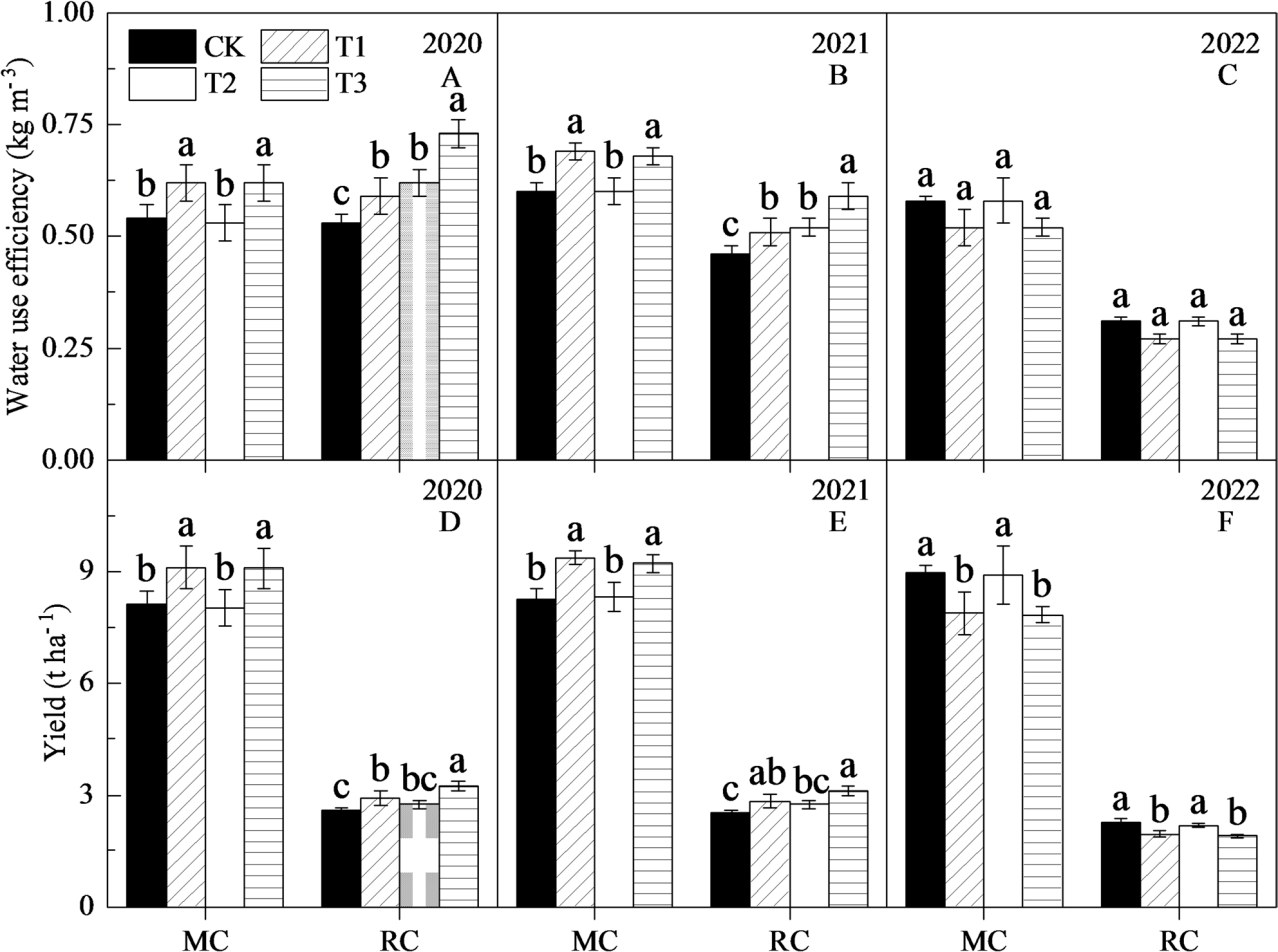
**A**-**F**. The impact of four irrigation treatments on the grain yield and water use efficiency of rice. CK, T1, T2 and T3 represent conventional irrigation, AWD irrigation during the filling period of main-season rice, AWD irrigation during the filling period of ratoon rice, and AWD irrigation during the filling period of both main-season rice and ratoon rice, respectively. MC and RC represent main-season rice and ratoon season rice, respectively. Values are means ± SE (*n* = 3). Different lowercase letters indicate significant differences among treatments within the same year and rice season (MC or RC) according to Duncan’s multiple range test (α = 0.05)

As shown in Fig. 10A-F, significant differences were observed in the WUE in the main-season rice as well as yield and WUE in the ratoon rice over different years. The order of WUE in the main-season rice was 2021 > 2020 > 2022. Compared with 2022, the WUE increased by 5.00% and 16.82% in 2020 and 2021, respectively. The order of yield and WUE in ratoon rice was 2020 > 2021 > 2022. Compared with 2022, the yield increased by 38.77% and 35.14%, and WUE increased by 117.70% and 79.65% in 2020 and 2021, respectively. In addition, the interaction of Y × T had a significant impact on yield and WUE. In 2020 and 2021, compared with CK, T1 and T3 exhibited an increased yield in the main-season rice by 12.81% and 11.66% and an increased WUE in the main-season rice by 14.90% and 14.07%, and T1, T2 and T3 exhibited an increased yield in the ratoon rice by 12.93%, 7.66% and 24.10% and an increased WUE in the ratoon rice by 11.10%, 15.01% and 33.00%, respectively. However, in contrast to 2020 and 2021, T1 and T3 in 2022 reduced rice yield and WUE. Comparison of CK in 2020 and 2021 with CK in 2022, T1 and T3 exhibited a decreased yield in the main-season rice by 12.04% and 12.60% and a decreased WUE in the main-season rice by 10.34% and 10.35%, respectively. T1 and T3 exhibited a decreased yield in the ratoon rice by 13.72% and 16.37% and a decreased WUE in the ratoon rice by 3.45% and 10.34%, respectively.

### Correlation analysis of soil and rice indicators

**Fig. 11:**
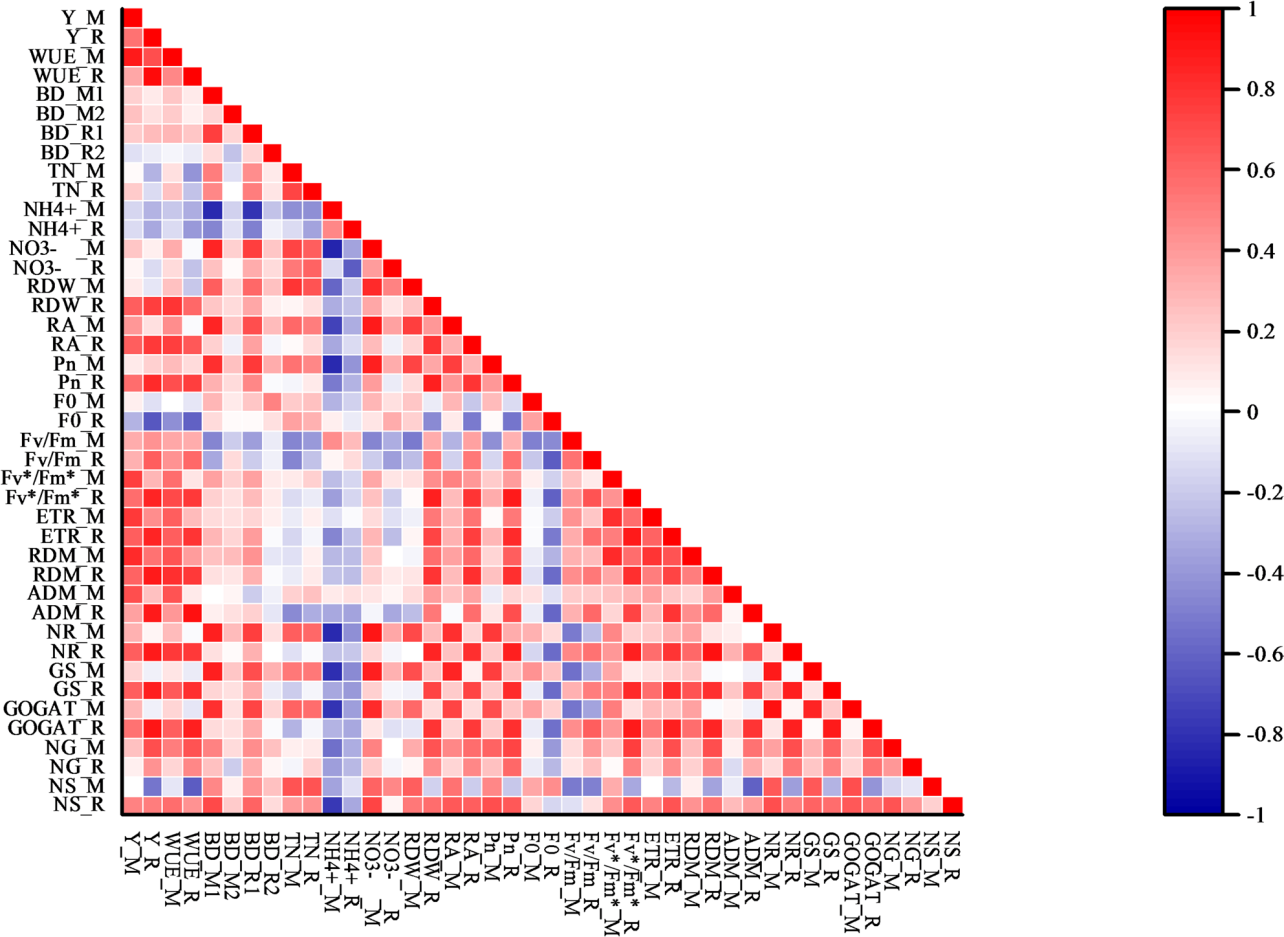
The correlation on Yield (Y), water use efficiency (WUE), soil bulk density (BD), soil total nitrogen (TN), NH_4_^+^ and NO_3_^−^ content. BD_M1 and BD_M2 represent 0 ~ 20 cm bulk density and 20 ~ 40 cm bulk density in main-season rice, and BD_R3 and BD_R4 represent 0 ~ 20 cm bulk density and 20 ~ 40 cm bulk density in ratoon rice, net photosynthetic rate (Pn), redistribution amount of dry matter stored at pre-anthesis from vegetative organs to grain (RDM), accumulation amount of dry matter at post-anthesis (ADM), minimum fluorescence (F0), maximal photochemical efficiency (Fv/Fm), actual photo chemical efficiency (Fv’/Fm’), electron transfer rate (ETR), activities of nitrate reductase (NR), glutamine synthetase (GS) and glutamate synthase (GOGAT), nitrogen concentration in grain (NG) and straw (NS). _R and _M represent main-season rice and ratoon rice, respectively

As shown in Fig. 11, the yield and WUE of the main-season rice showed a significant positive correlation with RA, RDM and ADM in the main-season rice. Additionally, the WUE of the main-season rice exhibited a significant positive correlation with the nitrogen concentration in the grains of the main-season rice. Conversely, the yield and WUE of ratoon rice were significantly negatively correlated with NH_4_^+^ concentration, whereas they showed a significant positive correlation with RDW, RA, Pn, RDM, ADM, nitrogen metabolism enzyme activity and nitrogen concentration in grains and straw during the ratoon season.

## Discussion

### Impact of irrigation management on soil characteristics

BD serves as a crucial indicator for assessing soil structure and suggesting soil permeability [35].In this study, AWD irrigation in the main-season rice (T1 and T3) significantly increased BD in the 0–20-cm soil layer. In the present study, AWD during the main season increased BD in the 0–20-cm soil layer. A likely mechanism is that repeated wetting–drying cycles under AWD caused consolidation and shrinkage of the puddled surface soil; because this structural change is not fully reversible after rewetting, total porosity decreases and soil strength and BD increase [36, 37].

Previous research has shown that elevated BD can restrict rice root growth and subsequently reduce yield [38]. Increased soil hardness physically restricts root penetration and elongation, limiting access to deeper soil layers [38]. Meanwhile, compacted soil diminishes oxygen diffusion and water infiltration, creating hypoxic conditions that impair root respiration and nutrient uptake [38]. In this study, although AWD irrigation increased BD, it did not reach the level of limiting yield under normal weather conditions. Additionally, the rice in this study was manually harvested. However, the trend in modern agriculture leans towards mechanised methods. In cases of mechanised harvesting for main-season rice, the crushing damage caused by the machine on the rice stubble seriously affects ratoon rice growth. AWD irrigation can enhance soil hardness and minimise machine-inflicted damage to the rice stubble [39].

The concentrations of TN, NH_4_^+^ and NO_3_^−^ indicate nitrogen levels and availability in soil. This study found that AWD irrigation in main-season rice (T1 and T3) increased TN concentration. This increase can be attributed to the fact that AWD irrigation reduces the amount of runoff, leakage and evaporation in the field, subsequently decreasing nitrogen loss [24]. Wang et al. [40] found that, compared with CK, AWD irrigation reduces leakage water by 22.5%, runoff water by 18.15%, N loss in leakage water by 27.1% and N loss in runoff water by 22.9%. Similarly, in this study, AWD irrigation reduced the irrigation water consumption by 4.9% and 23.1% for the main-season and ratoon rice, respectively. In this study, AWD irrigation in main-season rice (T1 and T3) reduced NH_4_^+^ concentration and increased NO_3_^−^ concentration, consistent with previous conclusions [41]. When rice paddies are flooded, soil respiration rapidly consumes oxygen in the soil, transitioning the soil from an aerobic environment to an anaerobic one [42]. Under anaerobic conditions, soil nitrification is significantly limited, resulting in NH_4_^+^ becoming the predominant form of nitrogen in the soil [34]. AWD irrigation increases oxygen availability, facilitating the conversion of NH_4_^+^ into NO_3_^−^ [41].

### Impact of irrigation management on yield and WUE of main-season rice

Improving rice yield and WUE is crucial to alleviate global food and water resource shortages [5]. Previous studies reported that AWD irrigation increased main-season rice yields in regenerative rice systems by 3.7% and 5.2% [23, 24]. In this study, it was observed that in 2020 and 2021, AWD irrigation in main-season rice (T1 and T3) increased various growth indicators, yield and WUE of main-season rice, aligning with previous findings. When soil is continuously flooded, soil respiration depletes oxygen, creating a hypoxic environment that reduces toxic substances in the rice rhizosphere and limits root growth [42]. AWD irrigation reduces these toxic substances, increases soil oxygen concentration and promotes the development of rice roots [43]. After reirrigation, the strong roots quickly absorb water and nutrients, further enhancing root and aboveground growth [8]. In addition, AWD irrigation reduces nutrient loss and increases TN concentration, thereby increasing RDW, plant nitrogen content and nitrogen metabolism enzyme activity. Similarly, Alhaj et al. [44] reported that, compared with CK, AWD irrigation increases the content of N, P and K in the soil. Generally, AWD reduced water-related nitrogen loss, increased soil TN and NO3−, and thereby may have improved nitrogen availability to rice plants. Song et al. [43] reported that AWD irrigation increased abscisic acid content in rice, stimulating the reactivation of prestored assimilates in stems and leaves, facilitating their transport to grains, and improving the filling efficiency of inferior spikelets. These findings align with this study, Under the normal-year conditions of this study, AWD irrigation slightly increased grain filling-related traits and improved yield formation. The reason may be that the AWD likely improved yield through enhanced nitrogen retention, root function, and assimilate redistribution. Under the hotter and drier conditions of 2022, however, the drying phase may have intensified physiological stress, limiting photosynthesis, nitrogen metabolism, and grain filling. Generally, the application of safe AWD irrigation during noncritical growth stages of crops can sustain or enhance rice yield, as observed not only in single- and double-cropping systems [12, 13] but also in ratoon rice systems under normal weather conditions, aligning with the findings in 2020 and 2021. The most striking finding of this study is the divergent impact of AWD irrigation on yields under contrasting weather conditions. In 2022, AWD irrigation in main-season rice (T1 and T3) reduced the 1000-grain RDM-yield and WUE of the main-season rice. This decline may be attributed to higher temperatures during rice heading to maturity in 2022 compared with 2020 and 2021 (35.8 °C vs. 33.6 and 32.8 °C). Previous studies indicate that temperatures exceeding 35 °C can adversely affect pollination, fertilisation and grain-filling processes in rice [6]. High temperatures can compromise the integrity of cell membranes, elevate reactive oxygen species levels, impair antioxidant enzyme activity, disrupt the photosynthetic system and alter the carbohydrate metabolism and distribution of photosynthates in rice [6]. Furthermore, this study found that under AWD irrigation, extreme heat and dryness in 2022 suppressed RA and net photosynthetic rate, hindering assimilate transport to grains, as indicated by reduced 1000-grain weight and low dry matter redistribution. The correlation analysis demonstrated that yield of main-season rice were significantly positively correlated with RA, RDM and ADM in the main-season rice. These findings suggest that high temperatures and drought likely reduced rice yield by impairing root development and activity, subsequently disrupting dry matter assimilation and transport, both crucial for grain filling. Notably, during soil drying phases under AWD irrigation in 2022, rice leaves displayed pronounced rolling, which subsided after reirrigation (data not displayed). In this study, we found that AWD irrigation treatment significantly reduced Fv/Fm, indicating abiotic stress on rice growth. This transient water deficit, compounded by high temperatures, likely disrupted stomatal regulation and carbon assimilation. Kong et al. [45] suggested that maintaining a 4–5-cm field water level during hot weather helps mitigate lipid peroxidation, improving rice photosynthesis and gas exchange and increasing rice yield. These studies are broadly consistent with our results. However, further research is required to delve into the underlying mechanisms of how AWD irrigation affects rice growth a

nd yield in high-temperature conditions.

### Impact of irrigation management on yield and WUE of ratoon rice

Ratoon rice yield constitutes one-third to two-thirds of the main-season rice yield, which can compensate for the main-season rice yield deficit caused by natural disasters and further stabilise food security [2]. Previous studies reported that AWD irrigation increased ratoon-season rice yields by 6.8% and 4.9% [23, 24]. In 2020 and 2021, the AWD irrigation in the main-season rice (T1 and T3) promoted increased various growth indicators, yield and WUE of the ratoon rice. These findings align with previous studies indicating that AWD irrigation enhances root development, photosynthesis, nitrogen metabolism and yield maintenance or improvement in ratoon rice [23–25]. A key reason that AWD irrigation in the main season enhanced ratoon rice growth was its ability to increase the TN and NO_3_^−^ levels in the soil during the growth period of ratoon rice, thereby facilitating N absorption. However, in 2022, the AWD irrigation in the main-season rice (T1 and T3) limited the growth, yield and WUE of the ratoon rice. Under the hotter and drier conditions of 2022, AWD likely intensified physiological stress by combining transient soil drying with heat stress, thereby suppressing root activity, photosynthetic performance, nitrogen metabolism, and assimilate transport to grain.The reason may be that the AWD irrigation exacerbates the damage of high temperatures and drought to the growth of main-season rice, which in turn affects ratoon rice growth. Meanwhile, the regeneration ability and subsequent growth of ratoon rice are closely linked to the functionality of the residual roots of the main-season rice and the new roots of the ratoon rice [3]. High-temperature stress results in increased death of residual roots and restricts the growth of new roots, particularly under the AWD irrigation. In addition, the mid-to-late stage of grain filling in main-season rice is a critical period for the germination and growth of axillary buds in ratoon rice [3]. AWD irrigation may exacerbate the high-temperature stress that inhibits the germination and growth of axillary buds, resulting in decreased Pn, hindered nitrogen metabolism, reduced grains per panicle and 1000-grain weight and reduced yield in ratoon rice. The correlation analysis results further corroborate our hypothesis that ratoon rice yield exhibits significant positive correlations with RDW, RA, Pn, RDM, ADM and nitrogen metabolism enzyme activity during the ratoon season.

The results of AWD irrigation during the ratoon rice season (T2) on the growth and yield of ratoon rice are consistent with those observed during the main season. In 2020 and 2021, AWD irrigation during the ratoon rice season increased various growth indicators, yield and WUE of the ratoon rice. However, in 2022, the trends reversed. This indicates that under high-temperature and drought conditions, AWD irrigation during the grain-filling period of ratoon rice (T2) limits its growth and yield. Hence, future research should focus on either developing rice varieties with enhanced tolerance to high temperature and drought stress or optimizing field management practices to mitigate these abiotic stresses. From a management perspective, AWD irrigation may be more suitable in ratoon rice under relatively stable weather conditions, whereas under hot and dry periods it should be combined with supplemental irrigation and heat-tolerant cultivars to reduce the risk of yield loss.

A limitation of this study is that it was conducted at a single site, and the climatic anomalies in 2022 co-occurred, particularly higher temperature and lower rainfall. Therefore, the divergent response to AWD irrigation in 2022 should be interpreted as the combined effect of multiple climatic stresses rather than temperature alone.

## Conclusion

This study presents the first field-based evidence of climate-dependent AWD irrigation effects in ratoon rice systems. Under normal weather (in 2020 and 2021), AWD irrigation enhanced yield and WUE by improving soil nitrogen availability and root function. In contrast, under the combined influence of hotter and drier conditions (2022), with possible interaction between heat stress and transient water deficit during AWD. AWD irrigation intensified yield losses due to suppressed RA, impaired nitrogen metabolism and disrupted assimilate partitioning. These findings fill a critical gap in AWD irrigation research, which has largely overlooked ratoon rice systems and natural heat waves. For practical implementation, we recommend that AWD irrigation adoption in ratoon rice be prioritised in areas with stable climates and integrate AWD irrigation with heat-tolerant varieties or supplemental irrigation during heat waves. By linking soil–plant interactions to climate variability, this study advances strategies for sustainable water management in a warming world.

## Data Availability

Data will be made available on request.

## Abbreviations

ADM: Accumulation amount of dry matter at post-anthesis
AK: Available Potassium
AN: Alkali-hydrolysed Nitrogen
AP: Available Phosphorus
AWD: Alternate Wetting and Drying irrigation
BD: Soil Bulk density
CK: Conventional irrigation
ETR: Electron Transfer Rate
F0: Minimum fluorescence
Fv/Fm: maximal photochemical efficiency
Fv’/Fm’: Actual photo chemical efficiency
GOGAT: Glutamate synthase
GS: Glutamine synthetase
MC: Main-season rice
NG: Nitrogen concentration in grain
NR: Activities of nitrate reductase
NS: Nitrogen concentration in straw
OM: Organic matter content
Pn: Net photosynthetic rate
RC: Ratoon season rice
RA: Root Activity
RDM: Redistribution amount of dry matter stored at pre-anthesis from vegetative organs to grain
RDW: Root Dry Weight
TK: Total potassium
TN: Total nitrogen
TP: total phosphorus
WUE: Water Use Efficiency

## References

[1] Firouzi S, Nikkhah A, Aminpanah H. Rice single cropping or ratooning agro-system: which one is more environment-friendly? Environ Sci Pollut Res Int. 2018;25:32246–56. 10.1007/s11356-018-3076-x. 30225691 10.1007/s11356-018-3076-x

[2] Wang W, He A, Jiang G, Sun H, Jiang M, Man J, Ling X, Cui K, Huang J, Peng S. Ratoon rice technology: A green and resource-efficient way for rice production. Adv Agron. 2020;159:135–67. 10.1016/BS.AGRON.2019.07.006.

[3] Xu F, Zhang L, Zhou X, Guo X, Zhu Y, Liu M, Xiong H, Jiang P. The ratoon rice system with high yield and high efficiency in China: Progress, trend of theory and technology. Field Crops Res. 2021;272:108282. 10.1016/j.fcr.2021.108282.

[4] Han J, Zhang Z, Luo Y, Cao J, Zhang L, Zhuang H, Cheng F, Zhang J, Tao F. Annual paddy rice planting area and cropping intensity datasets and their dynamics in the Asian monsoon region from 2000 to 2020. Agric Syst. 2022;200:103437. 10.1016/j.agsy.2022.103437.

[5] Huang W, Duan W, Chen Y. Rapidly declining surface and terrestrial water resources in Central Asia driven by socio-economic and climatic changes. Sci Total Environ. 2021;784:147193. 10.1016/j.scitotenv.2021.147193. 33905922 10.1016/j.scitotenv.2021.147193

[6] Xu Y, Chu C, Yao S. The impact of high-temperature stress on rice: Challenges and solutions. Crop J. 2021;9(5):963–76. 10.1016/j.cj.2021.02.011.

[7] Alauddin M, Sarker MAR, Islam Z, Tisdell C. Adoption of alternate wetting and drying (AWD) irrigation as a water-saving technology in Bangladesh: Economic and environmental considerations. Land Use Policy. 2020;91:104430. 10.1016/j.landusepol.2019.104430.

[8] Ishfaq M, Farooq M, Zulfiqar U, Hussain S, Akbar N, Nawaz A, Anjum SA. Alternate wetting and drying: A water-saving and ecofriendly rice production system. Agric Water Manage. 2020;241:106363. 10.1016/j.agwat.2020.106363.

[9] Islam SF, Sander BO, Quilty JR, De Neergaard A, Van Groenigen JW, Jensen LS. Mitigation of greenhouse gas emissions and reduced irrigation water use in rice production through water-saving irrigation scheduling, reduced tillage and fertiliser application strategies. Science of The Total Environment. 2020;739:140215. 10.1016/j.scitotenv.2020.140215. 10.1016/j.scitotenv.2020.140215 32758960

[10] Liang K, Zhong X, Fu Y, Hu X, Li M, Pan J, Liu Y, Hu R, Ye Q. Mitigation of environmental N pollution and greenhouse gas emission from double rice cropping system with a new alternate wetting and drying irrigation regime coupled with optimized N fertilization in South China. Agric Water Manage. 2023;282:108282. 10.1016/j.scitotenv.2020.140215.

[11] Wu Q, Gong F, Jia X, Tan M, Zhang W, Chi D. Maintaining rice grain yield under two irrigation regimes while reducing water-nitrogen input using acidified nitrogen-loaded biochar. Agric Water Manage. 2023;287:108432. 10.1016/j.agwat.2023.108432.

[12] Chu G, Chen T, Chen S, Xu C, Wang D, Zhang X. The effect of alternate wetting and severe drying irrigation on grain yield and water use efficiency of Indica-japonica hybrid rice (Oryza sativa L). Food Energy Secur. 2018;7(2):e133. 10.1002/fes3.133.

[13] Arai H. Increased rice yield and reduced greenhouse gas emissions through alternate wetting and drying in a triple-cropped rice field in the Mekong Delta. Sci Total Environ. 2022;842:156958. 10.1016/j.scitotenv.2022.156958. 35760167 10.1016/j.scitotenv.2022.156958

[14] Linquist B, Snyder R, Anderson F, Espino L, Inglese G, Marras S, Moratiel R, Mutters R, Nicolosi P, Rejmanek H, et al. Water balances and evapotranspiration in water- and dry-seeded rice systems. Irrig Sci. 2015;33(5):375–85. 10.1007/s00271-015-0474-4.

[15] Huda A, Gaihre YK, Islam MR, Singh U, Islam MR, Sanabria J, Satter MA, Afroz H, Halder A, Jahiruddin M. Floodwater ammonium, nitrogen use efficiency and rice yields with fertilizer deep placement and alternate wetting and drying under triple rice cropping systems. Nutr Cycl Agrosyst. 2016;104(1):53–66. 10.1007/s10705-015-9758-6.

[16] Pascual VJ, Wang Y. Utilizing rainfall and alternate wetting and drying irrigation for high water productivity in irrigated lowland paddy rice in southern Taiwan. Plant Prod Sci. 2017;20(1):24–35. 10.1080/1343943X.2016.1242373.

[17] Sriphirom P, Chidthaisong A, Towprayoon S. Effect of alternate wetting and drying water management on rice cultivation with low emissions and low water used during wet and dry season. J Clean Prod. 2019;223:980–8. 10.1016/j.jclepro.2019.03.212.

[18] Carrijo DR, Lundy ME, Linquist BA. Rice yields and water use under alternate wetting and drying irrigation: A meta-analysis. Field Crops Res. 2017;203:173–80. 10.1016/j.fcr.2016.12.002.

[19] Li G, Zhang J, Yang C, Song Y, Zheng C, Wang S, Liu Z, Ding Y. Optimal yield-related attributes of irrigated rice for high yield potential based on path analysis and stability analysis. Crop J. 2014;2(4):235–43. 10.1016/j.cj.2014.03.006.

[20] Sun Y, Xia G, He Z, Wu Q, Zheng J, Li Y, Wang Y, Chen T, Chi D. Zeolite amendment coupled with alternate wetting and drying to reduce nitrogen loss and enhance rice production. Field Crops Res. 2019;235:95–103. 10.1016/j.fcr.2019.03.004.

[21] Liang K, Zhong X, Huang N, Lampayan RM, Pan J, Tian K, Liu Y. Grain yield, water productivity and CH4 emission of irrigated rice in response to water management in south China. Agric Water Manage. 2016;163:319–31. 10.1016/j.agwat.2015.10.015.

[22] Jiang S, Du B, Hu F, Zhang H, Kong P, Wu Q, Zhu J. A seven-year study on the effects of four tillage modes on soil physicochemical properties, microbial biomass, enzymatic activities, and grain yield in a rice–ratoon rice cropping system. Food Energy Secur. 2023;12(2):e437. 10.1002/fes3.437.

[23] Zhang L, Jiang P, Gou X, Zhou X, Zhu Y, Liu M, Xiong H, Xu F. Integrated Water and Nitrogen Management Practices to Enhance Yield and Environmental Goals in Rice–Ratoon Rice Systems. Agron J. 2019;111(6):2821–31. 10.2134/agronj2018.10.0691.

[24] Ding Z, Li J, Hu R, Xiao D, Huang F, Peng S, Huang J, Li C, Hou J, Tian Y, et al. Root-zone fertilization of controlled-release urea reduces nitrous oxide emissions and ammonia volatilization under two irrigation practices in a ratoon rice field. Field Crops Res. 2022;287:108673. 10.1016/j.fcr.2022.108673.

[25] Leavitt M, Moreno-García B, Reavis CW, Reba ML, Runkle BRK. The effect of water management and ratoon rice cropping on methane emissions and yield in Arkansas. Agric Ecosyst Environ. 2023;356:108652. 10.1016/j.agee.2023.108652.

[26] Wang F, Cui K, Huang J. Progress and challenges of rice ratooning technology in Hubei Province, China. Crop Environ. 2023;2(1):12–6. 10.1016/j.crope.2023.02.002.

[27] Lu RK. Soil and agro-chemistry analytical methods [M][J]. 1999.

[28] YANG L, WANG Y, KOBAYASHI K, ZHU J, HUANG J, YANG H, WANG Y, DONG G, LIU G, HAN Y, et al. Seasonal changes in the effects of free-air CO₂ enrichment (FACE) on growth, morphology and physiology of rice root at three levels of nitrogen fertilization. Glob Change Biol. 2008;14(8):1844–53. 10.1111/j.1365-2486.2008.01624.x.

[29] Ramasamy S, Ten Berge HFM, Purushothaman S. Yield formation in rice in response to drainage and nitrogen application. Field Crops Res. 1997;51(1):65–82.

[30] Iqbal A, He L, Ali I, Ullah S, Khan A, Akhtar K, Wei S, Fahad S, Khan R, Jiang L. Co-incorporation of manure and inorganic fertilizer improves leaf physiological traits, rice production and soil functionality in a paddy field. Sci Rep. 2021;11(1):1–16. 10.1016/S0378-4290(96)01039-8. 33976273 10.1038/s41598-021-89246-9 PMC8113589

[31] Jiang S, Du B, Wu Q, Zhang H, Zhu J. Increasing pit-planting density of rice varieties with different panicle types to improves sink characteristics and rice yield under alternate wetting and drying irrigation. Food Energy Secur. 2021;12(1):e335. 10.1002/fes3.335.

[32] Shuochen J, Okpala NE, Lihe Z, Xiangru T, Bin D. Higher nitrogen application during rice growth increased yield and the biosynthesis of 2-acetyl-1-pyrroline under low light conditions. Field Crops Res. 2023;293:108846. 10.1016/j.fcr.2023.108846.

[33] Li D, Liu H, Qiao Y, Wang Y, Cai Z, Dong B, Shi C, Liu Y, Li X, Liu M. Effects of elevated CO2 on the growth, seed yield, and water use efficiency of soybean (Glycine max (L.) Merr.) under drought stress. Agric Water Manage. 2013;129:105–12. 10.1016/j.agwat.2013.07.014.

[34] Tan X, Shao D, Gu W. Effects of temperature and soil moisture on gross nitrification and denitrification rates of a Chinese lowland paddy field soil. Paddy Water Environ. 2018;16:687–98. 10.1007/s10333-018-0660-0.

[35] Rabot E, Wiesmeier M, Schlüter S, Vogel H. Soil structure as an indicator of soil functions: A review. Geoderma. 2018;314:122–37. 10.1016/j.geoderma.2017.11.009.

[36] Fang H, Zhou H, Norton GJ, Price AH, Raffan AC, Mooney SJ, Peng X, Hallett PD. Interaction between contrasting rice genotypes and soil physical conditions induced by hydraulic stresses typical of alternate wetting and drying irrigation of soil. Plant Soil. 2018;430(1–2):233–43. 10.1007/s11104-018-3715-5. 30147153 10.1007/s11104-018-3715-5 PMC6096897

[37] Fang H, Zhang Z, Li D, Liu K, Zhang K, Zhang W, Peng X, Zhou H. Temporal dynamics of paddy soil structure as affected by different fertilization strategies investigated with soil shrinkage curve. Soil Tillage Res. 2019;187:102–9. 10.1016/j.still.2018.12.006.

[38] Wang X, Qi JY, Liu BY, Kan ZR, Zhao X, Xiao XP, Zhang HL. Strategic tillage effects on soil properties and agricultural productivity in the paddies of Southern China. Land Degrad Dev. 2020;31(10):1277–86. 10.1002/ldr.3519.

[39] Zheng C, Wang Y, Yuan S, Xiao S, Sun Y, Huang J, Peng S. Heavy soil drying during mid-to-late grain filling stage of the main crop to reduce yield loss of the ratoon crop in a mechanized rice ratooning system. Crop J. 2022;10(1):280–5. 10.1016/j.cj.2021.06.003.

[40] Wang Y, Chen J, Sun Y, Jiao Y, Yang Y, Yuan X, Lærke PE, Wu Q, Chi D. Zeolite reduces N leaching and runoff loss while increasing rice yields under alternate wetting and drying irrigation regime. Agric Water Manage. 2023;277:108130. 10.1016/j.agwat.2022.108130.

[41] Li S, Chen Y, Li T, Yu F, Zhang Y, Liu K, Zhang H, Gu J, Yang J, Liu L. Alternate wetting and moderate soil drying irrigation counteracts the negative effects of lower nitrogen levels on rice yield. Plant Soil. 2022;481(1–2):367–84. 10.1007/s11104-022-05644-6.

[42] Bhaduri D, Mandal A, Chakraborty K, Chatterjee D, Dey R. Interlinked chemical-biological processes in anoxic waterlogged soil–A review. Indian J Agric Sci. 2017;87(12):1587–99. 10.56093/ijas.v87i12.76483.

[43] Song T, Xu F, Yuan W, Chen M, Hu Q, Tian Y, Zhang J, Xu W. Combining alternate wetting and drying irrigation with reduced phosphorus fertilizer application reduces water use and promotes phosphorus use efficiency without yield loss in rice plants. Agric Water Manage. 2019;223:105686. 10.1016/j.agwat.2019.105686.

[44] Alhaj Hamoud Y, Shaghaleh H, Sheteiwy M, Guo X, Elshaikh NA, Ullah Khan N, Oumarou A, Rahim SF. Impact of alternative wetting and soil drying and soil clay content on the morphological and physiological traits of rice roots and their relationships to yield and nutrient use-efficiency. Agric Water Manage. 2019;223:105706. 10.1016/j.agwat.2019.105706.

[45] Kong L, Ashraf U, Cheng S, Rao G, Mo Z, Tian H, Pan S, Tang X. Short-term water management at early filling stage improves early-season rice performance under high temperature stress in South China. Eur J Agron. 2017;90:117–26. 10.1016/j.eja.2017.07.006.

